# Co-receptor usage in distinct phylogenetic clusters of HIV-1 CRF01_AE is associated with differential immune reconstitution after antiretroviral therapy

**DOI:** 10.1128/spectrum.04222-25

**Published:** 2026-07-16

**Authors:** Yangyang Liu, Jing Lu, Tao Qiu, Zhiliang Hu, Wei Li, Xiaoshan Li, Guoping Du, Meng Zhao, Hongfei Ma, Pingmin Wei, Dandan Yang, You Ge

**Affiliations:** 1 Department of Infectious Disease, the Second Hospital of Nanjing, Nanjing University of Chinese Medicine 12579, Nanjing, Jiangsu, China; 2 Department of Epidemiology and Health Statistics, School of Public Health, Southeast University 66478, Nanjing, China; 3 Institute of HIV/AIDS/STI Prevention and Control, Jiangsu Provincial Center for Diseases Control and Prevention 12666, Nanjing, China; 4 Department of Quality Management, Children’s Hospital of Nanjing Medical University 159379, Nanjing, China; 5 Nanjing Medical University Affiliated Wuxi People's Hospital 261546, Wuxi, China; 6 Southeast University Hospital, Southeast University 12579, Nanjing, Jiangsu, China; University of Kentucky, Lexington, Kentucky, USA; San Raffaele Hospital, Milan, Italy; Aga Khan University, Karachi, Pakistan; Sun Yat-Sen University, Guangzhou, China

**Keywords:** HIV-1, CRF01_AE, cluster 4, immune reconstitution, co-receptor tropism

## Abstract

HIV-1 CRF01_AE cluster 4 is the predominant strain among men who have sex with men (MSM) in China and comprises two lineages, clusters 4a and 4b. We aimed to compare immune reconstitution after antiretroviral therapy (ART) between individuals infected with clusters 4a and 4b and to explore virological determinants underlying cluster-specific differences. We conducted a multicenter cohort study in Jiangsu Province, China, enrolling 604 cluster 4-infected patients with sustained viral suppression after 24 weeks of ART. We used generalized additive mixed models to characterize CD4^+^ T-cell recovery, logistic regression to identify factors associated with immunological non-response (INR), and Cox regression models to evaluate determinants of the CD4^+^ T-cell normalization. HIV-1 co-receptor tropism was inferred using Geno2pheno. We included 604 patients (391 cluster 4a, 213 cluster 4b). Patients with cluster 4a exhibited a significantly slower recovery rate than those with cluster 4b (*β*: −9.88 cells/μL/year; interaction term *P* = 0.006). Relative to cluster 4b, cluster 4a infection was associated with a higher risk of INR (adjusted OR: 1.61, 95% CI: [1.05, 2.48]) and a lower probability of CD4^+^ T-cell normalization (adjusted HR: 0.64, 95%CI: [0.49, 0.84]). Cluster 4a displayed a higher prevalence of CXCR4 tropism, driven by amino acid combinations at key V3 loop positions. Clusters 4a and 4b showed distinct patterns of immune reconstitution after ART. Cluster 4a infection was associated with poorer immune reconstitution, potentially related to its higher prevalence of CXCR4 tropism. Given the expanding prevalence of cluster 4 among MSM, monitoring its genetic evolution and virulence is crucial.

**IMPORTANCE:**

The differential impact of HIV-1 CRF01_AE clusters 4a and 4b on immune reconstitution after antiretroviral therapy (ART) remains unclear, particularly among men who have sex with men in China. Our study reveals that cluster 4a infection is associated with slower immune recovery and poorer clinical outcomes compared to cluster 4b, likely be related to its higher tendency for CXCR4 tropism. These findings highlight the need for vigilant monitoring of viral evolution and tailored management strategies for patients infected with distinct HIV-1 lineages.

## INTRODUCTION

CRF01_AE was the first known circulating recombinant form of HIV-1. Initially detected among people living with HIV-1 with heterosexual exposure and intravenous drug use (IDU) in the southwest provinces of China, CRF01_AE rapidly spread throughout the country (1–3). Previous evidence has suggested that CRF01_AE was more pathogenic than other subtypes in Asia. Specifically, CRF01_AE was associated with faster progression from estimated seroconversion to AIDS, higher plasma viral load, higher HIV-1 DNA level, faster CD4^+^ T-cell count loss, and shorter median survival (4–8). Despite sustained viral suppression under antiretroviral therapy (ART), CRF01_AE-infected patients were more likely to face suboptimal immune restoration (9–11).

The epidemic patterns of CRF01_AE in China were driven by multiple phylogenetic clusters(12). These distinct CRF01_AE clusters circulated among different high-risk populations. Clusters 1, 2, and 3 were prevalent among heterosexuals and IDU, whereas clusters 4 and 5 were primarily associated with men who have sex with men (MSM) (12, 13). Notably, these CRF01_AE clusters also exhibited divergent pathogenic characteristics. Previous studies reported distinct phenotypic evolution between clusters 4 and 5 despite both widely circulating among MSM populations (10, 14). Infection with cluster 4 was associated with more pronounced CD4^+^ T-cell depletion compared with cluster 5, and even under effective ART, individuals infected with cluster 4 showed poorer immune restoration than those infected with cluster 5.

Among these CRF01_AE clusters circulating in China, cluster 4 exhibits the highest genetic diversity and is uniquely divided into two well-supported sub-clusters, designated 4a and 4b, as confirmed by high-resolution phylogenetic analyses (12). Feng et al. first reported that cluster 4a comprised viruses exclusively from MSM in northern China, whereas cluster 4b contained strains sampled from non-MSM or individuals with unknown risk in eastern and southern provinces, indicating distinct underlying transmission networks within cluster 4 (12). More recent work by Dai et al. reported that individuals infected with clusters 4a and 4b typically presented with extremely low baseline CD4^+^ T-cell counts (median ~30–40 cells/µL) and a high prevalence of predicted CXCR4 tropism, features associated with more advanced disease and poorer immune prognosis (15). In the same setting, cluster 4b carried pretreatment drug-resistance mutations more frequently than 4a, especially V179D and S68G (15). Taken together, these findings indicate that clusters 4a and 4b already differ in their epidemiological and virological characteristics, raising the possibility that they may also diverge in clinical behavior, particularly with respect to immune reconstitution under ART. To address this knowledge gap, we conducted a retrospective longitudinal cohort study to compare immune reconstitution efficacy and virological features between patients infected with clusters 4a and 4b.

## RESULTS

### Characteristics of the included participants

We identified a total of 1,364 individuals infected with CRF01_AE. Phylogenetic analysis resolved these sequences into four clusters: cluster 4 (66.28%, 904/1,364), cluster 5 (20.09%, 274/1,364), cluster 1 (6.74%, 92/1,364), and cluster 2 (2.05%, 28/1,364). The remaining 61 sequences (4.84%) did not group into any established cluster (Fig. S1). Within cluster 4, further subdivision revealed two well-supported lineages, with cluster 4a accounting for 64.82% (586/904) and cluster 4b for 35.18% (318/904) of all the sequences (Fig. S1).

Patients were sequentially excluded for the following reasons: receiving ART <2 years (*N* = 24), lacking complete follow-up clinical data (*N* = 142), and failing to maintain sustained viral suppression after 24 weeks of ART (*N* = 134) (Fig. S2). A total of 604 individuals infected with cluster 4 (cluster 4a: 391; cluster 4b: 213) were ultimately included, contributing 2,768 person-years of follow-up. The median observation period was 4.21 years (IQR: 3.83, 5.04). Baseline characteristics of study participants were summarized in Table 1. CD4^+^ T-cell counts and age at ART initiation differed significantly between the cluster 4a and cluster 4b groups (*P* < 0.05 and SMD > 0.1). The gender distribution showed a borderline difference between the two clusters (*P* = 0.052). The majority of participants initiated ART with non-nucleoside reverse transcriptase inhibitor (NNRTI)-based regimens, specifically efavirenz (EFV, *n* = 547) or nevirapine (NVP, *n* = 50). Seven patients received regimens containing integrase strand transfer inhibitors (INSTIs), including dolutegravir (DTG, *n* = 4), elvitegravir (EVG, *n* = 2), and raltegravir (RAL, *n* = 1).

**TABLE 1:** Baseline characteristics between cluster 4a and cluster 4b patients at ART initiation

Variable	Classification	All patients			*P* value	SMD
		Overall (*N* = 604)	Cluster 4a (*N* = 391)	Cluster 4b (*N* = 213)		
Baseline age	–^*a*^	34 (27, 46)	35 (28, 47)	31 (26, 44)	0.029	0.187
	≤30	246 (40.73%)	146 (37.34%)	100 (46.95%)	0.040	0.218
	31–50	272 (45.03%)	182 (46.55%)	90 (42.25%)		
	>50	86 (14.24%)	63 (16.11%)	23 (10.80%)		
Baseline CD4 counts	–	272.00 (134.25, 405.75)	258.00 (107.00, 382.00)	314.00 (166.00, 442.50)	<0.001	0.291
	≤350 cells/µL	394 (65.23%)	273 (69.82%)	121 (56.81%)	0.001	0.273
	>350 cells/µL	210 (34.77%)	118 (30.18%)	92 (43.18%)		
Baseline BMI	<24	474 (78.48%)	312 (79.80%)	162 (76.06%)	0.285	0.090
	≥24	130 (21.52%)	79 (20.20%)	51 (23.94%)		
Baseline hemoglobin	<120 g/L	44 (7.28%)	29 (7.42%)	15 (7.04%)	0.304	0.129
	120–160 g/L	460 (76.16%)	304 (77.45%)	156 (73.24%)		
	>160 g/L	100 (16.56%)	58 (14.83%)	42 (19.72%)		
Time from HIV diagnosis to ART initiation	≤1 month	279 (46.19%)	185 (47.31%)	94 (44.13%)	0.506	0.064
	>1 month	325 (53.81%)	206 (52.69%)	119 (55.87%)		
Gender	Male	559 (92.55%)	360 (92.07%)	199 (93.43%)	0.544	0.052
	Female	45 (7.45%)	31 (7.93%)	14 (6.57%)		
HIV transmission route	HET	150 (24.83%)	92 (23.53%)	58 (27.23%)	0.315	0.085
	MSM	454 (75.17%)	299 (76.47%)	155 (72.77%)		
Marital status	Single	358 (59.27%)	227 (58.06%)	131 (61.50%)	0.410	0.070
	Married	246 (40.73%)	164 (41.94%)	82 (38.50%)		
Education background	Junior high school or lower	199 (32.95%)	123 (31.46%)	76 (35.68%)	0.224	0.149
	High school	170 (28.14%)	119 (30.43%)	51 (23.94%)		
	College or higher	235 (38.91%)	149 (38.11%)	86 (40.38%)		
History of STD	Yes	96 (15.89%)	59 (15.09%)	37 (17.37%)	0.464	0.062
	No/unknown	508 (84.11%)	332 (84.91%)	176 (82.63%)		
Initial antiretroviral regimen	NNRTIs-based	597 (98.84%)	385 (98.47%)	212 (99.53%)	0.284	0.101
	INSTIs-based	7 (1.16%)	6 (1.53%)	1 (0.47%)		

^*a*^ “–” indicates that the variable was analyzed as a continuous variable and therefore no classification was applicable.

### Slower recovery of CD4^+^ T-cell counts after ART in cluster 4a-infected patients

**Fig 1:**
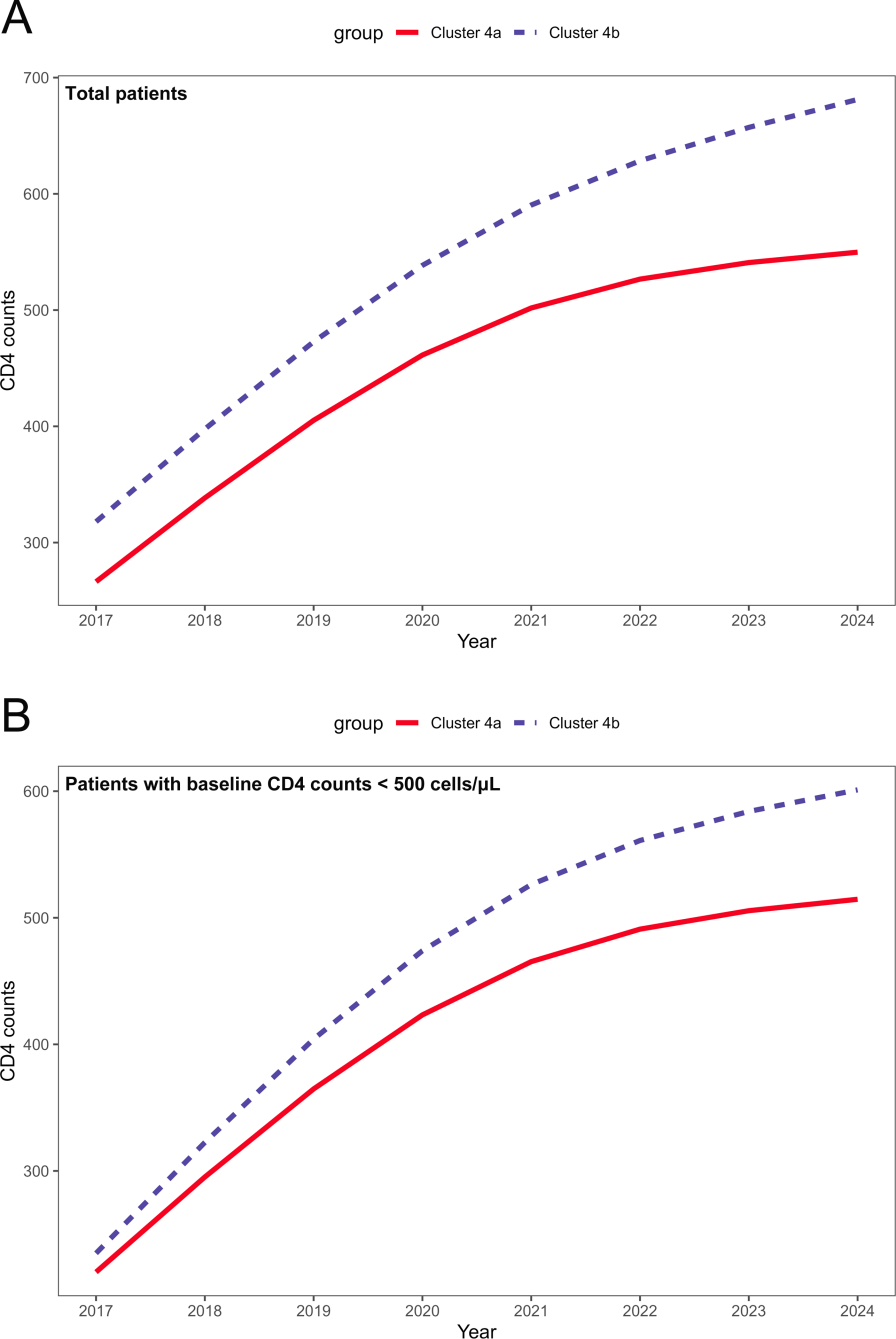
The smooth curve of CD4^+^ T-cell counts recovery fitted by generalized additive mixed model in total patients (**A**) and in patients with baseline CD4^+^ T-cell counts < 500 cells/µL (**B**).

In the GAMM analysis, both patients with clusters 4a and 4b showed significant annual increases in CD4^+^ T-cell counts following ART initiation (Cluster 4a, *β*: 46.15 cells/µL/year, 95% CI: [42.08, 50.21]; Cluster 4b, *β*: 55.91 cells/µL/year, 95% CI: [50.05, 61.77]) (Table 2). After adjustment for the covariates, patients with cluster 4a exhibited a significantly slower rate of CD4^+^ T-cell recovery compared with those with cluster 4b (*β*: −9.88 cells/µL/year, interaction term *P* = 0.006) (Table 2). Among patients with baseline CD4^+^ T-cell counts < 500 cells/µL, the slower recovery associated with cluster 4a remained significant (*β*: −8.12 cells/µL/year, interaction term *P* = 0.018) (Table 2). Figure 1A and B shows the GAMM-fitted smooth curves of CD4^+^ T-cell recovery.

**TABLE 2:** The analysis of annual recovery rate of CD4+ T-cell counts using GAMM^*b*^

Variable	All the patients (*N* = 604)		Patients with baseline CD4 cell counts < 500 cells/µL (*N* = 527)	
	Coefficient (95% CI)	*P* value^*a*^	Coefficient (95% CI)	*P* value^*a*^
Cluster 4b	55.91 (50.05, 61.77)	<0.001	55.17 (49.40, 60.94)	<0.001
Cluster 4a	46.15 (42.08, 50.21)	<0.001	47.23 (43.36, 51.10)	<0.001
Reference (Cluster 4b)	–^*d*^	–	–	–
Year* Cluster 4a (test for interaction)^*c*^	−9.88 (−16.89, −2.87)	0.006	−8.12 (−14.84, −1.41)	0.018

^*a*^ *P* value was adjusted by age at ART initiation, baseline CD4^+^ T-cell counts, baseline BMI, baseline hemoglobin, time from HIV diagnosis to ART initiation, gender, transmission route, marital status, education background, and STD history.

^*b*^ GAMM, generalized additive mixed model.

^*c*^ “*” indicates the interaction term between year and cluster group.

^*d*^ “–” indicates the reference group.

### Higher risk of immunological non-response in cluster 4a-infected patients

In the overall cohort, the proportion of INR was higher among patients with cluster 4a (43.73%) than among those with cluster 4b (28.64%). After adjusting for the covariates, cluster 4a remained independently associated with an increased risk of INR compared with cluster 4b (adjusted OR: 1.61, 95% CI: [1.05, 2.48], *P* = 0.029) (Table 3). A similar pattern was observed among individuals with baseline CD4^+^ T-cell counts < 500 cells/µL, where cluster 4a infected patients also showed a significantly higher risk of INR (adjusted OR: 1.59, 95% CI: [1.04, 2.45], *P* = 0.034) (Table 3).

**TABLE 3:** Differences in the risk of INR between cluster 4a and cluster 4b patients^*b*^

Variable	Classification	All patients (*N* = 604)				Patients with baseline CD4 counts < 500 cells/µL (*N* = 527)			
		INR (%)	*P* value	OR (95% CI)^*a*^	*P* value^*a*^	INR (%)	*P* value	OR (95% CI)^*a*^	P value^*a*^
CRF01_AE cluster 4	Cluster 4b	43.73	<0.001	–	–	48.44	0.004	–	–
	Cluster 4a	28.64		1.61 (1.05, 2.48)	0.029	35.06		1.59 (1.04, 2.45)	0.034
Baseline age	≤30	22.76	<0.001	–	–	27.72	<0.001	–	–
	31–50	46.69		2.03 (1.23, 3.35)	0.005	52.26		2.00 (1.21, 3.30)	0.007
	>50	56.98		2.53 (1.19, 5.37)	0.016	59.76		2.40 (1.13, 5.09)	0.023
Baseline CD4 counts	≤350 cells/µL	55.84	<0.001	–	–	55.84	<0.001	–	–
	>350 cells/µL	5.71		0.06 (0.03, 0.10)	<0.001	9.02		0.09 (0.05, 0.17)	<0.001
Baseline BMI	<24	39.87	0.358	–	–	45.22	0.280	–	–
	≥24	33.08		0.79 (0.48, 1.31)	0.365	39.45		0.79 (0.48, 1.30)	0.350
Baseline hemoglobin	<120 g/L	52.27	0.016	–	–	56.10	0.134	–	–
	120–160 g/L	39.35		0.83 (0.39, 1.74)	0.617	44.15		0.81 (0.38, 1.72)	0.588
	>160 g/L	28.00		0.72 (0.29, 1.80)	0.485	36.84		0.75 (0.30, 1.88)	0.541
Time from HIV diagnosis to ART initiation	≤1 month	42.65	0.047	–	–	46.12	0.341	–	–
	>1 month	34.77		1.28 (0.85, 1.92)	0.239	42.01		1.29 (0.86, 1.95)	0.210
Gender	Male	38.64	0.682	–	–	44.35	0.594	–	–
	Female	35.56		0.36 (0.15, 0.85)	0.019	40.00		0.35 (0.15, 0.85)	0.020
HIV transmission route	HET	42.00	0.297	–	–	48.09	0.279	–	–
	MSM	37.22		1.02 (0.59, 1.74)	0.954	42.68		1.00 (0.58, 1.73)	0.990
Marital status	Single	29.33	<0.001	–	–	34.43	<0.001	–	–
	Married	51.63		1.67 (1.04, 2.68)	0.034	57.21		1.71 (1.07. 2.75)	0.026
Education background	Junior high school or lower	48.24	<0.001	–	–	54.55	0.001	–	–
	High school	38.23		0.82 (0.48, 1.38)	0.448	44.30		0.80 (0.47, 1.36)	0.413
	College or higher	29.79		0.82 (0.47, 1.44)	0.488	34.65		0.80 (0.46, 1.41)	0.450
History of STD	Yes	34.48	0.375	–	–	38.82	0.292	–	–
	No/unknown	39.17		1.38 (0.79, 2.40)	0.253	45.02		1.38 (0.80, 2.41)	0.245
INSTI Switcher	No	37.89	0.286	–	–	48.48	0.594	–	–
	Yes	47.06		1.31 (0.57, 3.01)	0.523	43.72		1.29 (0.57, 2.96)	0.542

^*a*^ INR, immunological non-response, OR, Adjusted odds ratio.

^*b*^ “–” indicates the reference group.

### Lower probability of CD4 cell normalization in cluster 4a infected patients

**Fig 2:**
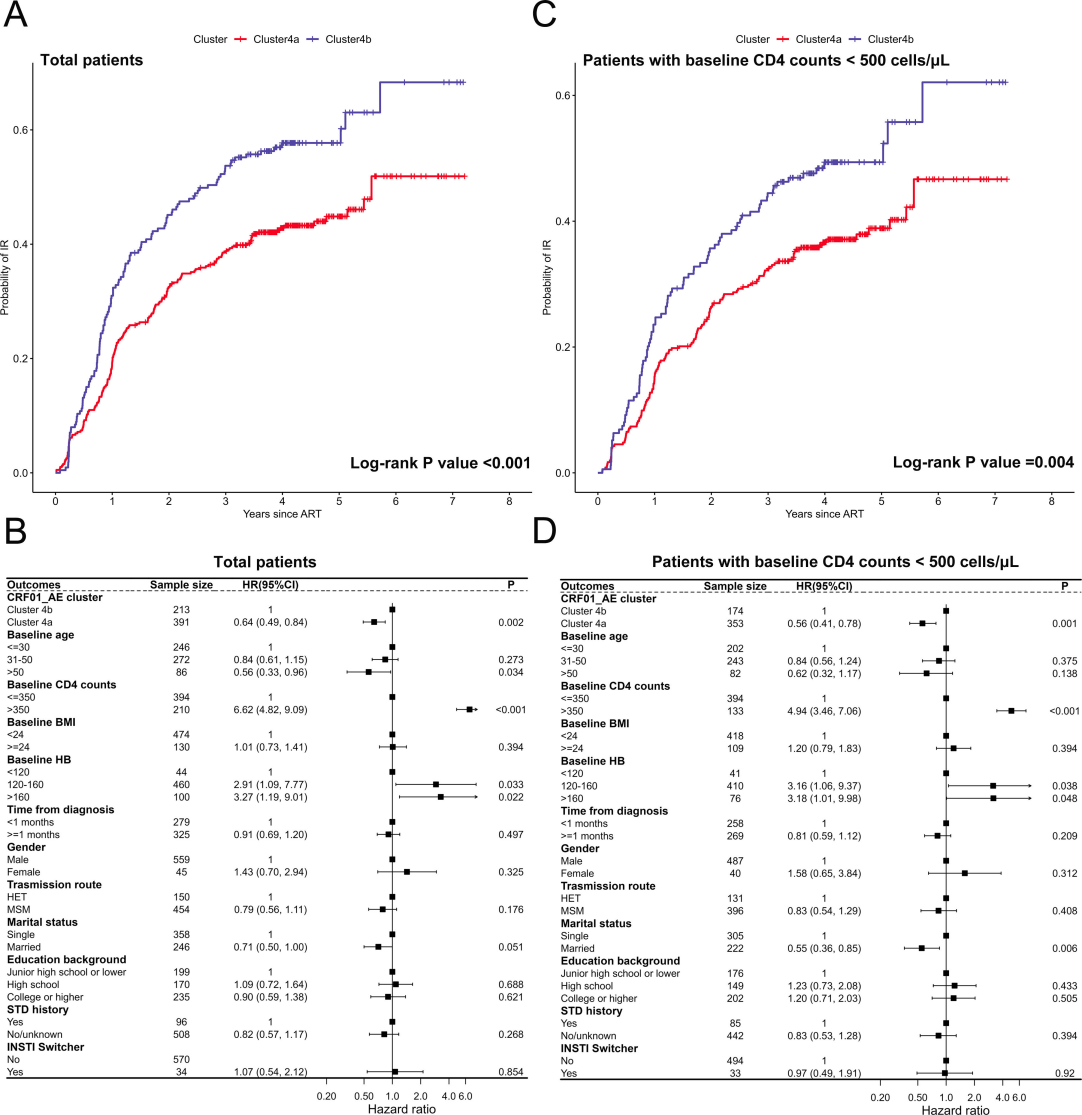
Comparison of the CD4 cell normalization probability between cluster 4a and cluster 4b patients. (**A**) The cumulative probability of achieving CD4 cell normalization in total patients. (**B**) The factors associated with CD4 cell normalization identified by multivariate time-dependent Cox regression analysis in total patients. (**C**) The cumulative probability of achieving CD4 cell normalization in patients with baseline CD4^+^ T-cell counts < 500 cells/µL. (**D**) The factors associated with CD4 cell normalization identified by multivariate time-dependent Cox regression analysis in patients with baseline CD4^+^ T-cell counts < 500 cells/µL.

The cumulative probability of CD4^+^ T-cell normalization increased over time in both groups, but cluster 4a-infected patients consistently showed a lower probability of CD4^+^ T-cell normalization compared with those infected with cluster 4b (Log-rank *P* < 0.001) (Fig. 2A). In multivariable time-dependent Cox regression analyses adjusting for potential confounders, with cluster 4b serving as the reference group (adjusted HR = 1.00), cluster 4a infection was associated with a lower probability of CD4^+^ T-cell normalization (adjusted HR: 0.64, 95% CI: [0.49, 0.84], *P* = 0.002) (Fig. 2B). A similar trend was observed among individuals with baseline CD4^+^ T-cell counts < 500 cells/µL (Fig. 2C and D), where cluster 4a infection was consistently linked to a lower probability of CD4^+^ T-cell normalization (adjusted HR: 0.56, 95% CI: [0.41, 0.78], *P* = 0.001), with cluster 4b as the reference group (adjusted HR = 1.00).

### Propensity score matching analysis

To address baseline imbalances in CD4^+^ T-cell counts (*P* < 0.001, SMD = 0.291) and age (*P* = 0.029, SMD = 0.187), a PSM analysis was performed. After 1:1 matching, the baseline CD4^+^ T-cell counts and age at ART initiation were well balanced between patients with clusters 4a and 4b (*P* = 0.958 and 0.713, respectively), with corresponding SMD values of 0.032 and 0.048, indicating excellent covariate balance (Table S1).

The trend of CD4^+^ T-cell counts recovery after ART was presented in Table S2 and Fig. S3. Similarly, CD4^+^ T-cell recovery in cluster 4a-infected patients appeared slower than that in those infected with cluster 4b (Total patients, interaction *β*: −13.68 cells/µL/year, *P* = 0.001; Patients with baseline CD4^+^ T-cell counts < 500 cells/µL, interaction *β*: −10.77, *P* = 0.002). Predictors associated with the risk of INR were summarized in Table S3. Infection with cluster 4a remained significantly associated with a higher risk of INR compared with cluster 4b (Total patients, adjusted OR: 1.95, 95% CI: [1.18, 3.23], *P* = 0.009; Patients with baseline CD4^+^ T-cell counts < 500 cells/µL, adjusted OR: 1.95, 95% CI: [1.17, 3.23], *P* = 0.010). The cumulative probability of achieving CD4 cell normalization was shown in Fig. S4. Consistent with the main findings, cluster 4a was associated with a lower probability of CD4 cell normalization (Total patients, adjusted HR: 0.73, 95% CI: [0.56, 0.95], *P* = 0.020; Patients with baseline CD4^+^ T-cell counts < 500 cells/µL, adjusted HR: 0.63, 95% CI: [0.46, 0.86], *P* = 0.004).

### Sensitivity analysis

Baseline plasma viral load at cART initiation is an important factor potentially influencing immune reconstitution. To further minimize confounding by baseline viral burden, we repeated all primary statistical analyses among the 355 participants with available baseline plasma viral load data, with baseline viral load additionally included as an adjustment covariate. The results remained consistent with the main analyses. After further adjustment for baseline viral load, cluster 4a infection was still associated with poorer immune reconstitution compared with cluster 4b infection (Tables S5 and S6 and Fig. S5).

### Higher prevalence of the CXCR4 tropism in cluster 4a

**Fig 3:**
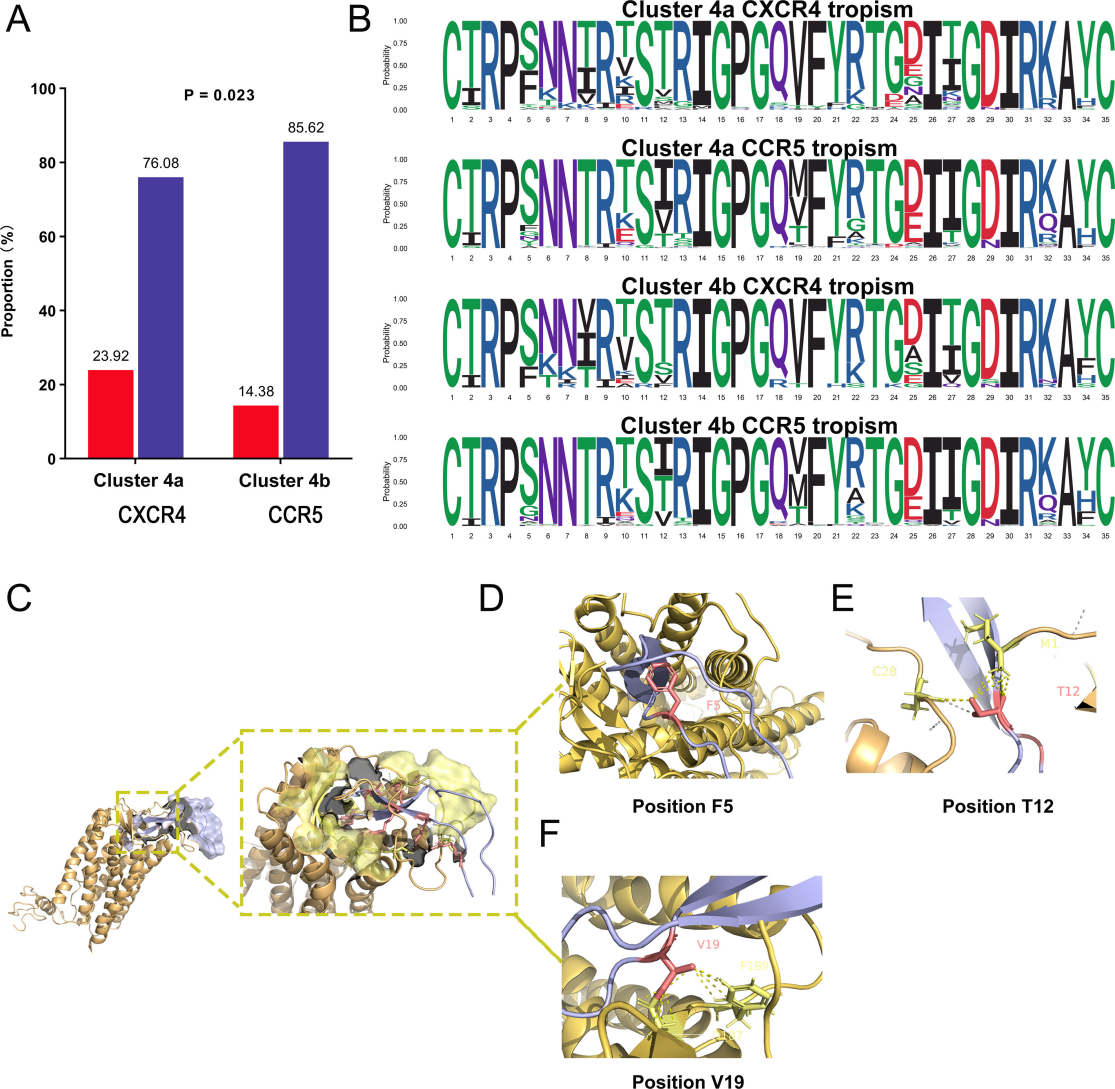
The tropism distribution and V3 loop structure of cluster 4a and cluster 4b. (**A**) Comparison of CXCR4 tropism distribution between cluster 4a and cluster 4b. Red represents CXCR4-tropic viruses, whereas blue represents CCR5-tropic viruses. (**B**) The frequency of the amino acids at V3 loop in CXCR4 and CCR5 tropic viruses of cluster 4a and cluster 4b. (**C**) The structural relationship between the V3 loop and the CXCR4 co-receptor docking. The light blue color represented the v3 loop, and the yellow-orange represented the CXCR4 co-receptor. (**D–F**) The action mode of the key amino acids at the V3 loop in the CXCR4 binding pocket, including F5 (**D**), T12 (**E**), and V19 (**F**).

HIV-1 tropism plays an important role in determining virulence. To explain the disparate impact of these two clusters on immune reconstitution, we analyzed HIV-1 tropism based on the V3 sequences of the env region. Among the 604 patients included in the immune reconstitution analysis, V3 sequences were successfully obtained from 401 individuals, comprising 255 from cluster 4a and 146 from cluster 4b. The proportion of CXCR4 tropism in cluster 4a was significantly higher than that in cluster 4b (cluster 4a: 23.92%, cluster 4b: 14.38%, *P* = 0.023) (Fig. 3A). We further evaluated the association between viral tropism and immune reconstitution among individuals with available V3 sequences. Patients harboring CXCR4-tropic viruses showed poorer immune reconstitution than those harboring CCR5-tropic viruses, suggesting that CXCR4 tropism may be associated with impaired immune reconstitution (Tables S7 and S8 and Fig. S6).

### Genetic properties of the V3 regions in cluster 4a and cluster 4b

To investigate the mechanisms underlying the higher propensity for CXCR4 tropism in cluster 4a, we compared the V3 sequences of these two sub-clusters. The V3 sequences of CXCR4-tropic viruses were largely comparable between clusters 4a and 4b. Unlike CCR5-tropic viruses, CXCR4-tropic viruses did not carry I at position 12 (HXB2 number: 307) or M at position 19 (HXB2 number: 314) (Fig. 3B). Instead, CXCR4-tropic variants frequently displayed the I-to-T substitution at position 12 and the V-to-M substitution at position 19. However, because these substitutions were also observed in a subset of CCR5-tropic sequences, additional amino acid changes might also be required for the shift from a CCR5- to a CXCR4-using phenotype. Previous studies have highlighted the importance of the N-linked glycan site and residue 25 in the V3 loop during the tropism switch in CRF01_AE strains (14). Among the V3 sequences analyzed, most CXCR4-tropic sequences showed loss of the N-linked glycan site at positions 6–8 (HXB2 number: 301–303), and non-E/D substitutions (S/A/G) at position 25 were enriched among CXCR4-using viruses. Notably, we also identified a novel and recurrent S-to-F substitution at position 5 (HXB2 number: 300), which was particularly common in CXCR4-tropic viruses from cluster 4a.

**Fig 4:**
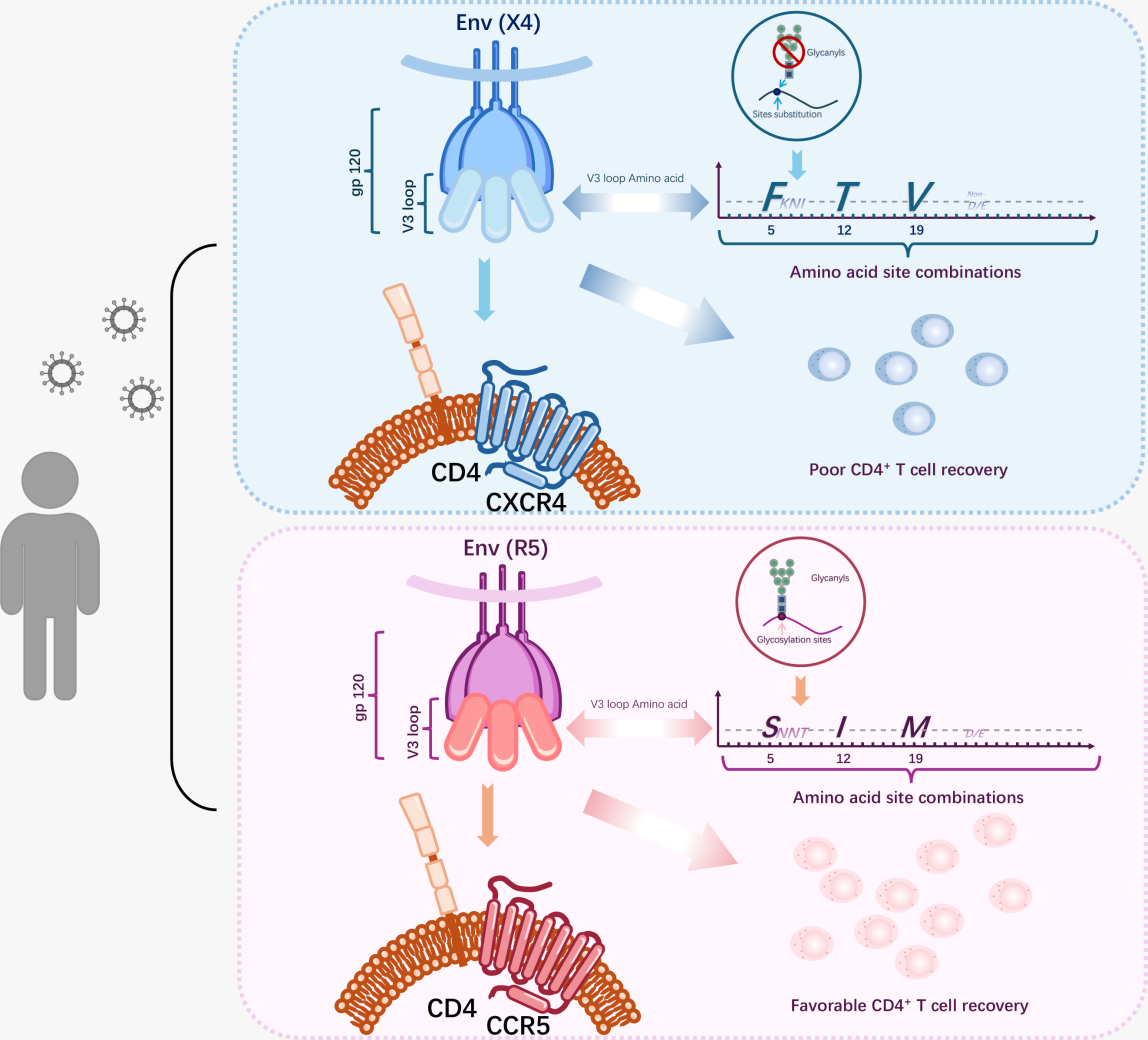
The schematic representation of the V3 loop structure of CXCR4- and CCR5-tropic viruses within CRF01_AE cluster 4.

Subsequently, we performed structural modeling by simulating the docking of the V3 loop with the CXCR4 co-receptor (Fig. 3C). We focused on three newly identified and previously uncharacterized residues in the V3 loop (F5, T12, and V19). In the V3–CXCR4 docking model, F5 was classified as a hydrophobic residue whose side chain was folded inward toward the core of the V3 loop, without forming direct contacts with CXCR4 residues, consistent with the typical burial of hydrophobic side chains within the protein structure (Fig. 3D). Since the residue T12 in the V3 loop and residue C28 in the CXCR4 receptor were both hydrophilic residues, T12 was more compatible with the hydrophilic microenvironment at the V3–CXCR4-binding interface (Fig. 3E). V19 in the V3 loop, a hydrophobic residue positioned within the CXCR4 ligand-binding pocket, formed non-hydrogen bonding forces with D187 and F189 of CXCR4 (Fig. 3F). Taken together, these structural features suggest that the combination of specific amino acid alterations at key positions within the V3 loop may contribute to the CXCR4 tropism shift, which was more frequently observed in cluster 4a (Fig. 4).

## DISCUSSION

In this multicenter cohort of individuals infected with CRF01_AE cluster 4, we systematically compared post-ART immune reconstitution between clusters 4a and 4b. We found that cluster 4a infection was associated with a slower increase in CD4^+^ T-cell counts, a higher risk of INR, and a lower probability of CD4 cell normalization. Following PSM to adjust for potential confounding bias by matching on baseline CD4^+^ T-cell count and baseline age, two key determinants of immune reconstitution, the study results remained robust. Previous studies have highlighted pathogenic differences between CRF01_AE clusters, particularly the more severe clinical and immunological outcomes associated with cluster 4 compared with cluster 5 (10, 14, 16). However, no prior study has directly compared the patterns of immune recovery among patients infected with the two sub-clusters within cluster 4. To our knowledge, this study is the first to demonstrate that the two sub-clusters within cluster 4 represent biologically distinct lineages that may be associated with different patterns of immune reconstitution after ART.

One plausible explanation for the impaired immune reconstitution observed in cluster 4a infection is the markedly higher prevalence of predicted CXCR4 tropism. The entry into target cells for HIV-1 mainly depends on the binding of the virus to the CD4-receptor and chemokine co-receptors CCR5 or CXCR4 (17). The transmitted and founder viruses with CCR5 tropism have selective transmission advantages during early infection, whereas the switch from CCR5 to CXCR4 tropism is often detected at later disease stages (18–20). In CRF01_AE infections, most co-receptor switches occur within 3 years after infection, much sooner than the time required for other subtypes such as subtypes B and C (19, 21–23). The emergence of CXCR4 tropism is associated with chronic immune activation, accelerated disease progression, and poor immunological response to ART (20, 24–28).

In our sequence analysis, cluster 4a exhibited a unique pattern of amino acid substitutions at key positions within the V3 loop, the primary determinant for CCR5 or CXCR4 tropism (29). Shao et al. have revealed the association of amino acid substitutions at specific positions in the V3 loop with CXCR4 tropism in CRF01_AE cluster 4 (10, 14). However, our results indicated that the combination of amino acid substitutions at specific positions may be associated with CXCR4 tropism in cluster 4. First, the non-I 12 and non-M 19 were the common features in all the CXCR4 tropic sequences. On this basis, the V3 loop of CXCR4 tropic viruses could lose the N-linked glycan site or harbor non-E/D25 substitutions or F5 substitutions. The proportion of this combination of amino acid alterations in clusters 4a and 4b was 20.39% and 12.33%, respectively, which may partly account for the higher prevalence of CXCR4 tropism in cluster 4a. Taken together, these structural features in the V3 loop may contribute to the CXCR4 tropism shift in cluster 4a and provide a potential mechanistic basis for its poorer immunological outcomes.

Given the widespread prevalence of cluster 4 in MSM, continued monitoring of its genetic evolution and potential clinical implications is important. The poorer immune reconstitution associated with cluster 4a suggests that this lineage may contribute to long-term adverse immunological outcomes despite viral suppression, highlighting the need for earlier diagnosis, closer post-treatment monitoring, and timely interventions for affected individuals. Moreover, incorporating tropism signatures into routine surveillance and clinical assessment may improve risk stratification and help identify patients at higher risk of incomplete immune reconstitution.

This study had potential limitations. First, baseline plasma viral load data at ART initiation were not available for all participants. Although additional sensitivity analyses among participants with available baseline viral load data showed that the main findings remained unchanged after further adjustment for baseline viral load, residual confounding related to baseline viral load cannot be completely excluded. Second, HIV-1 co-receptor usage was predicted from the env V3-coding region using the Geno2pheno platform, as recommended by the European Consensus Group on the clinical management of HIV-1 tropism testing. Although an FPR threshold of 2% has been validated in China for distinguishing CRF01_AE tropism, employing the phenotypic tropism assay would enable a more accurate evaluation. Third, although we performed exploratory structural analyses using homology modeling and molecular docking to compare the predicted interactions between cluster-specific V3 structures and the CXCR4/CCR5 co-receptors, these computational analyses cannot replace functional validation. Functional assays would be valuable for determining whether the sequence differences observed in cluster 4a directly contribute to altered co-receptor usage. In addition, molecular dynamics simulations were not performed; such analyses would provide further information on the dynamic stability of receptor–ligand interactions and binding energy changes over time. These functional and computational validation approaches should be incorporated in future studies to further clarify the molecular basis of the observed epidemiological associations. Finally, as a retrospective cohort study, our analysis could identify associations but could not establish causality. In particular, although cluster 4a showed a higher prevalence of CXCR4 tropism and CXCR4 tropism was associated with poorer immune reconstitution, we could not formally determine whether viral tropism mediated the association between cluster 4a infection and impaired immune reconstitution. Further prospective studies incorporating mediation analysis should be performed to validate the potential pathway.

### Conclusions

In this multicenter cohort, the two major sub-clusters within CRF01_AE cluster 4 showed different patterns of immune reconstitution after ART. Cluster 4a infection was associated with poorer immune reconstitution than cluster 4b infection. Cluster 4a had a higher prevalence of predicted CXCR4-tropic variants, which may represent a potential explanatory factor for its poorer immune reconstitution, although this hypothesis requires further validation. Given the large epidemic size of cluster 4 among MSM, ongoing surveillance of its genetic evolution and potential changes in virulence is crucial.

## MATERIALS AND METHODS

### Study participants

We conducted a multicenter retrospective cohort study across the Centers for Disease Control and Prevention in 13 cities in Jiangsu Province, China. The study population comprised individuals aged ≥18 years who were newly diagnosed with HIV infection between 2017 and 2018. Individuals were eligible if they met the following criteria: (i) infection with CRF01_AE clusters 4a or 4b; (ii) receipt of ART for at least 2 years; (iii) availability of complete post-ART CD4^+^ T-cell follow-up records; and (iv) achievement of virological suppression (HIV-1 RNA < 50 copies/mL) after 24 weeks of ART. The follow-up period began at ART initiation and ended in December 2024. All participants underwent free annual monitoring of CD4^+^ T-cell counts and plasma HIV-1 RNA levels as part of routine care. The study was designed according to the STROBE guidelines. This study was approved by the ethical review board of the Jiangsu Provincial Center for Diseases Control and Prevention (JSJK2022-B001-02). The study was conducted in accordance with the Declaration of Helsinki (1964) and its later amendments.

### Data source

The demographic information was extracted from the China National HIV/AIDS Comprehensive Response Information Management System, including age, gender, HIV transmission route, marital status, educational background, and history of sexually transmitted diseases (STD). Clinical data were obtained from the national database established under China’s National Free Antiretroviral Treatment Program in late 2004 (30). These data included the dates of HIV diagnosis and ART initiation; ART regimen; baseline CD4^+^T-cell counts, HIV-1 viral load, BMI, and hemoglobin levels at ART initiation; and all subsequent CD4^+^T-cell counts and viral load measurements during follow-up.

### CRF01_AE clusters identification and co-receptor tropism prediction

As previously described (31), HIV-1 RNA was extracted from 200 μL of plasma using the Abbott Sample Preparation System (Abbott Molecular, Inc., Des Plaines, IL). Reverse-transcription PCR followed by nested PCR was performed to amplify the pol region (HXB2: 2,253–3,312 nt) and the env region (HXB2: 7,002–7,541 nt). The specific primer sequences used for the amplification of the pol and env regions are detailed in Table S4. The RT-PCR was performed with the following thermal cycling conditions: 50°C for 30 min, 94°C for 5 min, 55°C for 1 min, and 68°C for 2 min, followed by 40 cycles of 94°C for 30 s, 55°C for 45 s, and 68°C for 1 min 30 s, with a final extension at 68°C for 10 min and an indefinite hold at 4°C. The nPCR was conducted under the following conditions: an initial denaturation at 94°C for 2 min, 55°C for 1 min, and 72°C for 1 min 30 s, followed by 40 cycles of 94°C for 30 s, 55°C for 45 s, and 72°C for 1 min 30 s. The reaction was concluded with a final extension at 72°C for 10 min and an indefinite hold at 4°C.

HIV-1 genotypes were determined through phylogenetic analysis. Reference sequences representing different CRF01_AE clusters were obtained from a published study (12). An approximate maximum-likelihood phylogenetic tree was constructed using FastTree v2.2 to identify distinct CRF01_AE clusters. The Generalized Time-Reversible model with gamma-distributed rate variation and a proportion of invariable sites (GTR + G + I) was selected as the nucleotide substitution model. The resulting phylogenetic tree was visualized and annotated utilizing FigTree v1.4.4. The criteria for defining the CRF01_AE clusters were as follows: (i) number of sequences in a cluster ≥20 and (ii) bootstrap value ≥0.70. HIV-1 co-receptor usage was inferred from the env V3 loop using the Geno2pheno platform. Consistent with previous studies on CRF01_AE tropism in China, a false-positive rate (FPR) threshold of 2% was applied to define CXCR4 tropism (14). This cutoff has been experimentally validated and shown to provide high accuracy for tropism inference in epidemiological settings.

### Structural model building

Consensus V3 sequences, inherently incorporating the specific cluster-defining mutations for 4a and 4b, were generated prior to modeling. The tertiary structures of the HIV-1 env V3 loops were predicted via homology modeling using the SWISS-MODEL workspace, with the env-gp120 protein structure (PDB ID: 6CK9) as a template (32). Model quality was rigorously verified using the platform’s built-in assessment tools. The generated 3D models demonstrated high structural reliability, achieving 100% sequence coverage and 80% target-template sequence identity. Overall structural stability was further confirmed by a Global Model Quality Estimation (GMQE) score of 0.80 and a QMEAN score of 0.72 ± 0.12, justifying their use in downstream docking analyses.

To simulate receptor interactions, structural templates for the host co-receptors were separately prepared. The full-length CXCR4 structure was constructed by combining previously reported structures (PDB IDs: 3OE0 and 2K05) (33, 34). PDB-2K05 contains the 35 N-terminal amino acid residues that are missing in PDB-3OE0. Therefore, we aligned PDB-2K05 with PDB-3OE0 using PyMOL and then reconstructed the missing N-terminal residues in PDB-3OE0. Molecular Graphics System. Conversely, the structural template for the CCR5 co-receptor was derived from its established crystal structure (PDB ID: 4MBS). Use AutoDockTools to prepare the target receptor and V3 loop structures by removing water molecules, adding polar hydrogens, and assigning partial charges. The binding pocket was then defined in AutoDock Vina, with the grid box centered at coordinates (*x* = −5.443, *y* = 0.286, *z* = −4.107) and box dimensions set to 70 × 82 × 70 Å, followed by molecular docking calculations.

### Immune reconstitution definitions

We evaluated the immune reconstitution efficacy through (i) CD4^+^ T-cell counts increase rate; (ii) risk of immunological non-response (INR); and (iii) time to CD4^+^ T-cell normalization. INR was defined as persistent CD4^+^ T-cell counts < 500 cells/µL after 3 years of treatment; CD4^+^ T-cell normalization, as two consecutive CD4^+^ T-cell counts ≥ 500 cells/µL after virological suppression (35, 36).

### Statistical analysis

Demographic and clinical characteristics were compared between CRF01_AE clusters using chi-square tests for categorical variables and Kruskal-Wallis tests for continuous variables. We fitted generalized additive mixed models (GAMMs) with random intercepts and random slopes to characterize longitudinal recovery of CD4^+^ T-cell counts after ART initiation. Interaction terms between CRF01_AE clusters (cluster 4a vs cluster 4b) and the time effect were included to compare cluster-specific rates of CD4^+^ T-cell recovery. Logistic regression models were used to assess the association of CRF01_AE clusters with the risk of INR. Kaplan-Meier analysis and time-dependent Cox proportional hazards models were employed to compare time to CD4^+^ T-cell normalization between clusters 4a and 4b infected patients. Based on prior clinical knowledge and previously reported predictors of immune reconstitution (37, 38), a predefined set of covariates considered to potentially influence CD4^+^ T-cell recovery was included in all multivariable models using a forced-entry approach. These confounding factors included age, gender, marital status, transmission route, baseline CD4^+^ T-cell counts, baseline BMI, baseline hemoglobin, history of STD, and duration from HIV-1 diagnosis to starting ART.

As the patients with baseline CD4^+^ T-cell counts ≥ 500 cells/µL might not experience incomplete immune reconstitution, those patients would introduce biases in our results. Therefore, we repeated all analyses used in this study in the subgroup of patients with baseline CD4^+^ T-cell counts < 500 cells/µL. To minimize baseline imbalances between individuals infected with cluster 4a and cluster 4b, we performed the propensity score matching (PSM). Propensity scores were estimated using a logistic regression model with CRF01_AE cluster 4 subgroups as the dependent variable and baseline CD4^+^ T-cell count and age at ART initiation as covariates. These two variables were included because they showed substantive baseline imbalance between cluster 4a and cluster 4b before matching (*P* < 0.05 and standardized mean difference [SMD] > 0.1). One-to-one nearest-neighbor matching without replacement was then performed. All subsequent analyses were repeated in the matched cohort to evaluate the robustness of the findings.

Statistical significance was defined as *P* < 0.05. All statistical analyses were performed using R (version 4.0.4). PSM was performed using the MatchIt package. Survival analyses, including Kaplan–Meier analysis and time-dependent Cox models, were performed using the survival package. GAMM was fitted using the mgcv package. Logistic regression analyses were performed using the base R stats package.

## Supplementary Material

Reviewer comments

## Data Availability

The sequence data generated in this study have been deposited in figshare and are publicly available at the following DOI: https://doi.org/10.6084/m9.figshare.25018643.

## SUPPLEMENTAL MATERIAL

Supplemental figures and tables spectrum.04222-25-s0001.docx Tables S1 to S8 and Figure S1 to S6.

OPEN PEER REVIEW reviewer-comments.pdf An accounting of the reviewer comments and feedback.

ASM does not own the copyrights to Supplemental Material that may be linked to, or accessed through, an article. The authors have granted ASM a non-exclusive, world-wide license to publish the Supplemental Material files. Please contact the corresponding author directly for reuse.
