# Supplementary material for: Co-receptor usage in distinct phylogenetic clusters of HIV-1 CRF01_AE is associated with differential immune reconstitution after antiretroviral therapy
Source: Microbiol Spectr. 2026 Jul 16;14(8):e04222-25. doi: 10.1128/spectrum.04222-25 (PMC13436356; doi:10.1128/spectrum.04222-25)
Supplement: Supplementary file 1 [file reviewer-comments.pdf]

AMERICAN SOCIETY FOR MICROBIOLOGY | Microbiology Spectrum

# Co-receptor usage in distinct phylogenetic clusters of HIV-1 CRF01_AE is associated with differential immune reconstitution after antiretroviral therapy

Yangyang Liu, Jing Lu, Tao Qiu, Zhiliang Hu, Wei Li, Xiaoshan Li, Guoping Du, Meng Zhao, Hongfei Ma, Pingmin Wei, Dandan Yang, and You Ge

*Corresponding Author(s): Pingmin Wei, Southeast University*

**Review Timeline:**

| | |
|---|---|
| Submission Date: | January 3, 2026 |
| Editorial Decision: | March 15, 2026 |
| Revision Received: | May 13, 2026 |
| Accepted: | June 6, 2026 |

*Editor: Jaime Soria*

*Reviewer(s): Disclosure of reviewer identity is with reference to reviewer comments included in decision letter(s). The following individuals involved in review of your submission have agreed to reveal their identity: Priscilla Biswas (Reviewer #1); Syed Hani Hassan Abidi (Reviewer #2); Jinfeng Cai (Reviewer #3)*

## Transaction Report:

(Note: With the exception of the correction of typographical or spelling errors that could be a source of ambiguity, letters and reports are not edited. The original formatting of letters and referee reports may not be reflected in this compilation.)

**DOI: https://doi.org/10.1128/spectrum.04222-25**

Re: Spectrum04222-25 (**Co-receptor usage in distinct phylogenetic clusters of HIV-1 CRF01_AE is associated with differential immune reconstitution after antiretroviral therapy**)

Dear Prof. Pingmin Wei:

Thank you for the privilege of reviewing your work. Below you will find my comments, instructions from the Spectrum editorial office, and the reviewer comments.

Please return the manuscript within 60 days; if you cannot complete the modification within this time period, please contact me. If you do not wish to modify the manuscript and prefer to submit it to another journal, notify me immediately so that the manuscript may be formally withdrawn from consideration by Spectrum.

**Revision Guidelines**
To submit your modified manuscript, log into the submission site at https://spectrum.msubmit.net/cgi-bin/main.plex. Go to Author Tasks and click the appropriate manuscript title to begin. The information you entered when you first submitted the paper will be displayed; update this as necessary. Note the following requirements:

• Upload point-by-point responses to the issues raised by the reviewers in a file named "Response to Reviewers," NOT in your cover letter.
• Upload a compare copy of the manuscript (without figures) as a "Marked-Up Manuscript" file.
• Upload a clean .DOC/.DOCX version of the revised manuscript and remove the previous version.
• Each figure must be uploaded as a separate, editable, high-resolution file (TIFF or EPS preferred), and any multipanel figures must be assembled into one file.
• Any supplemental material intended for posting by ASM should be uploaded with their legends separate from the main manuscript. You can combine all supplemental material into one file (preferred) or split it into a maximum of 10 files with all associated legends included.

For complete guidelines on revision requirements, see our Submission and Review Process webpage. Submission of a paper that does not conform to guidelines may delay acceptance of your manuscript.

**Data availability:** ASM policy requires that data be available to the public upon online posting of the article, so please verify all links to sequence records, if present, and make sure that each number retrieves the full record of the data. If a new accession number is not linked or a link is broken, provide Spectrum production staff with the correct URL for the record. If the accession numbers for new data are not publicly accessible before the expected online posting of the article, publication may be delayed; please contact production staff (Spectrum@asmusa.org) immediately with the expected release date.

**Publication Fees:** For information on publication fees and which article types are subject to charges, visit our website. If your manuscript is accepted for publication and any fees apply, you will be contacted separately about payment during the production process; please follow the instructions in that e-mail. Arrangements for payment must be made before your article is published.

**ASM Membership:** Corresponding authors may join or renew ASM membership to obtain discounts on publication fees. Need to upgrade your membership level? Please contact Customer Service at Service@asmusa.org.

The ASM Journals program strives for constant improvement in our submission and publication process. Please tell us how we can improve your experience by taking this quick Author Survey.

Thank you for submitting your paper to Spectrum.

Sincerely,
Jaime Soria
Editor
Microbiology Spectrum

Reviewer #1 (Comments for the Author):

The paper is very clearly written. The initial antiretroviral regimen is mainly NNRTI-based, I would only suggest to specify which NNRTI drugs are used and to write what is INSTI (integrase - based?).

Reviewer #2 (Comments for the Author):

Liu et al. investigated how HIV-1 CRF01_AE clusters 4a and 4b differently affect immune recovery following antiretroviral therapy. This study enhances knowledge of the sub-clusters' roles in immune restoration and their contribution to disease progression.

Nonetheless, there are multiple areas for improvement and some sections that lack sufficient detail. The authors need to address the comments below to clarify their work and improve their manuscript.

• Line 35: Give the total number of patients
• Line 68: When you say more pathogenic, can you also describe what is known about CRF01_AE-mediated disease progression rate, symptoms, co-morbidities, etc
• Line 87: Statement about genetic diversity needs to be supported by a reference. What is the reported genetic diversity for this cluster?
• Line 123: Describe the ART therapy they were on? Was there a change in therapy during follow-up? If yes, was that accounted for when calculating INR or immune recovery?
• Line 125: Gender was also different (just reaching significance level, p=0.05), creating the possibility for biological differences in recovery. This should also be discussed here and in the discussion
• Line 156: Also give HR for cluster b
• Line 163: This should also be done for gender
• Table 3: Are the ORs shown here adjusted or unadjusted?
• Figure 3: What are the purple and red colors showing in part A?
• Line 352: 353: Mention the PCR primers and conditions used for pol and env amplification
• Line 356: How was the ML tree constructed? Also, give model details and parameters used
• Line 363: Statement needs to be supported by a reference.
• Line 365: More details about protein modeling and insertion of mutations are required. How was model quality verified? How were mutations inserted and then validated for stability, quality score, coverage, etc?
• Line 372: Was the docking performed as described in the results? The methods say nothing about how docked poses were generated. Are details about dock pose generation and interaction required? Also, if docking was done, it would be very interesting to perform MD simulations on the docked pose (you can limit it to just 100 ns) to see changes in binding energy over time. This will help explain how substitutions are contributing to changes in receptor-protein interactions.
• Line 390: Was it a multi-variable model and step-wise?
• Line 393: Statement needs to be supported by a reference. Also, how was the adjustment done?
• Line 406: Define and describe details for PSM model
• Line 411: Using what packages?

Reviewer #3 (Comments for the Author):

Major Comments
1.The absence of baseline HIV RNA levels represents a major limitation that directly affects interpretation of immune recovery. Baseline viral load is one of the strongest predictors of CD4 recovery after ART and may differ substantially between viral lineages. Without adjustment for this parameter, it remains unclear whether the observed differences between cluster 4a and 4b reflect viral pathogenicity or differences in disease stage at ART initiation. Although the authors acknowledge this limitation, the impact is likely underestimated. At present, the central conclusion that cluster 4a is intrinsically more pathogenic remains insufficiently supported.

2.The authors propose that the poorer immune recovery in cluster 4a is "likely driven" by higher CXCR4 tropism. However, CXCR4 tropism was not included in multivariable models, mediation analysis was not performed, direct association between tropism and CD4 recovery was not shown, thus the claim that tropism explains immune recovery differences is currently speculative. To strengthen this central hypothesis, some level of experimental validation is strongly encouraged.

3. The manuscript repeatedly states that cluster 4a has "greater pathogenic potential" or is "more virulent." However, the study only demonstrates the differences in immune reconstitution after ART, it does not evaluate disease progression pre-ART/mortality/AIDS events/viral replication capacity. The authors should revise language throughout the manuscript.

This manuscript by Liu et al. investigates the differential immune reconstitution following antiretroviral therapy (ART) among individuals infected with HIV-1 CRF01_AE cluster 4a versus 4b in Jiangsu Province, China. Using a multicenter longitudinal cohort, the authors demonstrate that cluster 4a infection is associated with slower $CD4^{+}$ T-cell recovery, higher risk of immunological non-response (INR), and lower probability of CD4 normalization. The study further suggests that a higher prevalence of CXCR4 tropism in cluster 4a may partly explain these differences. Overall, the study is well organized and provides potentially novel epidemiological insights. However, the manuscript in its current form remains primarily descriptive, and several methodological and mechanistic issues limit the strength of the conclusions. Substantial revisions are required before the manuscript is suitable for publication.

Major Comments

1. The absence of baseline HIV RNA levels represents a major limitation that directly affects interpretation of immune recovery. Baseline viral load is one of the strongest predictors of CD4 recovery after ART and may differ substantially between viral lineages. Without adjustment for this parameter, it remains unclear whether the observed differences between cluster 4a and 4b reflect viral pathogenicity or differences in disease stage at ART initiation. Although the authors acknowledge this limitation, the impact is likely underestimated. At present, the central conclusion that cluster 4a is intrinsically more pathogenic remains insufficiently supported.

2. The authors propose that the poorer immune recovery in cluster 4a is "likely driven" by higher CXCR4 tropism. However, CXCR4 tropism was not included in multivariable models, mediation analysis was not performed, direct association between tropism and CD4 recovery was not shown, thus the claim that tropism explains immune recovery differences is currently speculative. To strengthen this central hypothesis, some level of experimental validation is strongly encouraged.

3. The manuscript repeatedly states that cluster 4a has "greater pathogenic potential" or is "more virulent." However, the study only demonstrates the differences in immune reconstitution after ART, it does not evaluate disease progression pre-ART/mortality/AIDS events/viral replication capacity. The authors should revise language throughout the manuscript.

**Response to Reviewer's comments**

**We sincerely appreciate the time and effort the reviewers and editors spent to review our manuscript. We are grateful for the constructive comments, which have greatly improved the quality and clarity of the work. We have carefully revised the manuscript in accordance with the reviewers' suggestions and provide a detailed, point-by-point response below. For the editors' and reviewers' convenience, we have submitted a marked-up version of the revised manuscript, in which all changes are highlighted in yellow.**

**Reviewer #1**

**Comment 1:** The paper is very clearly written. The initial antiretroviral regimen is mainly NNRTI-based, I would only suggest to specify which NNRTI drugs are used and to write what is INSTI (integrase - based?).

**Response:** Thank you for your valuable comments. We have revised the "Characteristics of the included participants" subsection in the Results section to specify the NNRTI and INSTI drugs used in the initial antiretroviral regimens and to define INSTI as integrase strand transfer inhibitor **(Page 4, Lines 123–128)**. INSTIs are a class of antiretroviral drugs that prevent viral DNA from integrating into the host cell genome by blocking HIV integrase.

The revised text is as follows: **The majority of participants initiated ART with non-nucleoside reverse transcriptase inhibitor (NNRTI)-based regimens, specifically efavirenz (EFV, n = 547) or nevirapine (NVP, n = 50). Seven patients received regimens containing integrase strand transfer inhibitors (INSTIs), including dolutegravir (DTG, n = 4), elvitegravir (EVG, n = 2), and raltegravir (RAL, n = 1)**.

---

**Reviewer #2**

**Comment 1:** Line 35: Give the total number of patients

**Response:** Thank you for your thoughtful reminder. We have added the total number of patients included in the analysis to the Abstract section **(Page 2, Line 35)**.

**Comment 2:** Line 68: When you say more pathogenic, can you also describe what is known about CRF01_AE-mediated disease progression rate, symptoms, co-morbidities, etc

**Response:** Thank you for your constructive suggestion. In the original manuscript, the term "more pathogenic" was used to refer to previously reported virological, immunological, and clinical indicators of poorer outcomes associated with CRF01_AE infection, including higher plasma viral load, higher HIV-1 DNA levels, faster $CD4^+$ T-cell decline, shorter median survival, and a higher likelihood of suboptimal immune restoration despite sustained viral suppression under ART.

We agree that the description of CRF01_AE-associated disease progression was not sufficiently detailed. We have therefore added evidence regarding the progression rate reported by Li et al., who showed that CRF01_AE infection was associated with faster progression from estimated seroconversion to AIDS compared with non-CRF01_AE subtypes (4.8 vs. 6.4 years). The available evidence mainly reflects disease progression, virological burden, immune deterioration, and immune recovery after ART, rather than subtype-specific clinical symptoms or comorbidities. Although a few studies have reported associations between CRF01_AE infection and certain opportunistic infections, such as extrapulmonary cryptococcosis, these findings are more likely to reflect the consequences of more rapid immune deterioration and advanced immunodeficiency rather than CRF01_AE-specific clinical manifestations.

Accordingly, we have revised the relevant text in the Introduction section and added the corresponding references **(Page 3, Line 67-69).** The revised text is as follows: **Previous evidence has suggested that CRF01_AE was more pathogenic than other subtypes in Asia. Specifically, CRF01_AE infection was associated with faster progression from estimated seroconversion to AIDS, higher plasma viral load, higher HIV-1 DNA levels, faster $CD4^+$ T-cell decline, and shorter median survival. Despite sustained viral suppression under antiretroviral therapy (ART), CRF01_AE-infected patients were also more likely to experience suboptimal immune restoration.**

**Comment 3.** Line 87: Statement about genetic diversity needs to be supported by a reference. What is the reported genetic diversity for this cluster?

**Response:** Thank you for your careful observation. We realize that the statement in the original manuscript was too brief and lacked the necessary epidemiological background and literature support. Since CRF01_AE entered the MSM population in China in the early 1990s, Feng et al. (2013) identified seven CRF01_AE circulating clusters (including 4a and 4b) based on near-full-length genome sequences. According to the latest nomenclature in 2024, eight CRF01_AE clusters are currently confirmed to be circulating in China. Among these eight distinct clusters, only Cluster 4 exhibits uniquely high genetic diversity evolutionarily, which directly led to its further differentiation into two distinct sub-lineages (Cluster 4a and 4b), while the remaining seven clusters did not show a similar tendency for sub-lineage differentiation.

We have added the relevant reference to support the statement regarding the genetic diversity of cluster 4 and revised the corresponding text as follows: **Among these CRF01_AE clusters circulating in China, cluster 4 exhibits the highest genetic diversity and is uniquely divided into two well-supported sub-clusters, designated 4a and 4b, as confirmed by high-resolution phylogenetic analyses (Page 3, Line 84-86).**

**Comment 4.** Line 123: Describe the ART therapy they were on? Was there a change in therapy during follow-up? If yes, was that accounted for when calculating INR or immune recovery?

**Response:** Thank you for pointing out this important clinical detail. We acknowledge that the ART regimens and treatment changes during follow-up were not sufficiently described in the original manuscript. We have made the following improvements:

First, we have added detailed information on the ART regimens in the Results section **(Page 4, Lines 123–128)**. **In our cohort, the vast majority of patients (597 cases) initiated NNRTI-based regimens, specifically including efavirenz (EFV, n = 547) and nevirapine (NVP, n = 50). A very small number of patients (7 cases) received INSTI-based regimens, including dolutegravir (DTG, n = 4), elvitegravir (EVG, n = 2), and raltegravir (RAL, n = 1). During follow-up, 34 patients switched from NNRTI-based regimens to INSTI-based regimens.**

Second, in order to account for treatment switching when calculating INR, we included switching from an NNRTI-based regimen to an INSTI-based regimen during follow-up as an additional covariate in the logistic regression analysis for INR. This treatment switch was not significantly associated with INR ($P = 0.286$). After adjustment for this covariate, cluster 4a remained associated with a higher risk of INR than cluster 4b **(see**

**Revised Table 3)**.

Third, in order to account for treatment switching when evaluating $CD4^+$ T-cell normalization, we performed a time-dependent Cox regression analysis by including "INSTI switcher" as a time-dependent covariate. After adjustment for treatment switching, the main results remained unchanged: patients infected with cluster 4a had a lower probability of achieving $CD4^+$ T-cell normalization than those infected with cluster 4b **(see Revised Figure 2)**.

In addition, treatment switching was further adjusted for in the PSM analysis. The association between cluster 4a infection and poorer immune reconstitution remained consistent. The updated PSM analysis results are provided in the Supplementary Materials **(see Revised Supplementary Table S3 and Figure S4)**.

**Comment 5.** Line 125: Gender was also different (just reaching significance level, P=0.05), creating the possibility for biological differences in recovery. This should also be discussed here and in the discussion.

**Response:** Thank you for your detailed review of the baseline data. We agree that gender is a potential confounding factor that may influence immune reconstitution, especially as previous studies have suggested better immune reconstitution in females than in males. In our cohort, the vast majority of patients in both clusters were male. Although the difference in gender distribution was borderline significant, the proportion of female patients was 7.45% in cluster 4a and 6.57% in cluster 4b, indicating that the absolute difference between the two clusters was modest. More importantly, gender was included as a covariate in all multivariable analyses. After adjustment for gender and other potential confounders, cluster 4a remained associated with poorer immune reconstitution than cluster 4b, supporting the robustness of our main findings. We have added the following sentence to the Results section: "The gender distribution showed a borderline difference between the two clusters (P = 0.052) **(Page 4, Lines 123-124)**.

**Comment 6**. Line 156: Also give HR for cluster b

**Response:** Thank you for your reminder. In our time-dependent Cox regression analysis, cluster 4b was defined as the reference group; therefore, its HR was 1.00 by definition. After adjustment for potential confounders, the adjusted HR (aHR) for cluster 4a compared with cluster 4b was 0.64. This indicates that patients infected with cluster 4a showed slower immune recovery than those infected with cluster 4b. To improve clarity and explicitly indicate the reference group, we have added the HR for cluster 4b (reference group, HR = 1.00) to the main text **(Page 5, Lines 157-163)**.

**Comment 7.** Line 163: This should also be done for gender

**Response:** Thank you for your suggestion. We agree that gender may influence immune reconstitution and should be properly considered in the analysis. In this study, gender was included as an adjustment covariate in all multivariable models, together with other clinically relevant factors such as age and baseline CD4+ T-cell count, to control for potential confounding.

Regarding a separate gender-stratified analysis, we carefully considered this suggestion. However, the sample size of female participants in our cohort was limited compared with male participants (45 vs. 559), which would result in insufficient statistical power and unstable estimates, especially for subgroup analyses stratified by both gender and CRF01_AE sub-cluster. In addition, the primary objective of this study was to evaluate the independent association between CRF01_AE cluster 4a/4b and immune reconstitution, whereas gender was treated as an important potential confounder rather than a primary exposure of interest. Therefore, we retained gender as an adjustment factor in the multivariable analyses rather than conducting additional gender-stratified analyses.

**Comment 8.** Table 3: Are the ORs shown here adjusted or unadjusted?

**Response:** Thank you for raising this question. The OR shown in Table 3 are the adjusted odds ratio, as indicated by the column heading "aOR." To avoid any ambiguity, we have revised the table footnote to explicitly define aOR as adjusted odds ratio. Thank you again for helping us improve the clarity and transparency of our reporting.

**Comment 9.** Figure 3: What are the purple and red colors showing in part A?

**Response:** We sincerely apologize for the unclear color annotation in Figure 3A. In this panel, red represents CXCR4-tropic viruses, whereas blue represents CCR5-tropic viruses. To improve clarity, we have added a visual legend directly to **Revised Figure 3A** and updated the figure legend accordingly. The revised legend now reads: **(A) Comparison of CXCR4 tropism distribution between cluster 4a and cluster 4b. Red represents CXCR4-tropic viruses, whereas blue represents CCR5-tropic viruses.**

**Comment 10.** Line 352: 353: Mention the PCR primers and conditions used for pol and env amplification

**Response:** Thank you for pointing out this important omission. To improve the reproducibility of our study, we have supplemented the primer information and PCR conditions used for pol and env amplification.

Specifically, we added **Supplementary Table S4**, which lists the primer sequences used in the first- and second-round amplification reactions. We also revised the Methods section to include the thermal cycling conditions for the first-round RT-PCR and the second-round nested PCR. The newly added text is as follows:

**The specific primer sequences used for the amplification of the pol and env regions are detailed in Supplementary Table S4. The RT-PCR was performed with the following thermal cycling conditions: 50°C for 30 min, 94°C for 5 min, 55°C for 1 min, and 68°C for 2 min, followed by 40 cycles of 94°C for 30 s, 55°C for 45 s, and 68°C for 1 min 30 s, with a final extension at 68°C for 10 min and an indefinite hold at 4°C. The nPCR was conducted under the following conditions: an initial denaturation at 94°C for 2 min, 55°C for 1 min, and 72°C for 1 min 30 s, followed by 40 cycles of 94°C for 30 s, 55°C for 45 s, and 72°C for 1 min 30 s. The reaction was concluded with a final extension at 72°C for 10 min and an indefinite hold at 4°C (Page 10, Lines374-383).**

**Comment 11.** Line 356: How was the ML tree constructed? Also, give model details and parameters used

**Response:** Thank you for your helpful reminder. We have revised the Methods section to provide additional details on phylogenetic tree construction, including the software, evolutionary model, and visualization tool used **(Page 10, Lines 387-391).**

Specifically, we clarified that an approximate maximum-likelihood phylogenetic tree was constructed using FastTree v2.2. The generalized time-reversible model with gamma-distributed rate variation and a proportion of invariant sites (GTR+G+I) was used as the nucleotide substitution model. The resulting tree was visualized using FigTree v1.4.4. The revised text is as follows: **An approximate maximum-likelihood (ML) phylogenetic tree was constructed using FastTree v2.2 to identify distinct CRF01_AE clusters. The Generalized Time-Reversible model with gamma-distributed rate variation and a proportion of invariable sites (GTR+G+I) was selected as the nucleotide substitution model. The resulting phylogenetic tree was visualized and annotated utilizing FigTree v1.4.4**

**Comment 12.** Line 363: Statement needs to be supported by a reference.

**Response:** We thank you for pointing out this omission. We have now added the appropriate reference [**Tamamis P, Floudas CA. 2014. Molecular Recognition of CCR5 by an HIV-1 gp120 V3 Loop. PLOS One 9:e95767**.] to correctly support the statement regarding the initial structural template (PDB ID: 6CK9) used for the CCR5 conformation **(Page 11, Lines 405).**

**Comment 13.** Line 365: More details about protein modeling and insertion of mutations are required. How was model quality verified? How were mutations inserted and then validated for stability, quality score, coverage, etc?

**Response:** Thank you for your questions regarding the methodological details of protein modeling. We would like to clarify that the amino acid differences analyzed in this study were not manually inserted into pre-existing three-dimensional structures. Instead, they were already present in the cluster-specific V3 consensus sequences generated for clusters 4a and 4b in FASTA format. These sequences were then submitted separately to SWISS-MODEL for homology modeling. SWISS-MODEL automatically identifies suitable structural templates and evaluates the resulting models based on parameters including target–template sequence identity, coverage, and the Global Model Quality Estimate (GMQE). In our analysis, the generated models showed a target–template sequence identity of 80%, coverage of 100%, and a GMQE score of 0.80. These parameters support the reliability of the homology models for comparative structural analysis.

We have revised and reorganized the "Structural model building" subsection in the Methods section to provide additional methodological details regarding protein modeling and molecular docking procedures **(Page 10-11, Lines 399-423).**

**Comment 14.** Line 372: Was the docking performed as described in the results? The methods say nothing about how docked poses were generated. Are details about dock pose generation and interaction required? Also, if docking was done, it would be very interesting to perform MD simulations on the docked pose (you can limit it to just 100 ns) to see changes in binding energy over time. This will help explain how substitutions are contributing to changes in receptor-protein interactions.

**Response:** Thank you very much for this highly inspiring and professional suggestion.

First, regarding the methodological details of molecular docking, we apologize for the omission in the original Methods section. The docking analysis was performed as described in the Results, but the details of docked pose generation and interaction analysis were not sufficiently provided. We have revised and reorganized the "Structural model building" subsection in the Methods section to provide additional methodological details regarding protein modeling and molecular docking procedures **(Page 10-11, Lines 399-423)**. Specifically, we added information on the docking software used (AutoDock Vina), the receptor and ligand preparation process, the center coordinates and dimension settings of the Grid box, and the criteria for selecting the optimal conformation based on the lowest binding affinity.

Second, regarding MD simulations, we agree that molecular dynamics (MD)

simulations of the docked complexes would provide additional information on the dynamic stability of receptor–ligand interactions and binding energy changes over time, and a 100-ns MD simulation would strengthen the mechanistic interpretation of the docking results. However, in the current study, our structural analysis was intended as an exploratory approach to provide preliminary structural support for the observed differences in CXCR4 tropism between cluster 4a and cluster 4b. Therefore, we focused primarily on homology modeling and molecular docking analyses. Although MD simulations would further strengthen the mechanistic interpretation, they are beyond the scope of the current revision. Therefore, we have now added a statement in the Discussion section acknowledging that the absence of MD simulations is a limitation of the present study. We also note that future studies incorporating MD simulations and binding free-energy analyses will be important for further validating the dynamic stability and interaction differences observed in our docking analyses **(Page 8-9, Lines 311-321)**.

**Comment 15.** Line 390: Was it a multi-variable model and step-wise?

**Response:** Thank you for your comment. The analyses were performed using multivariable regression models rather than stepwise regression procedures. Specifically, CRF01_AE cluster 4 subgroups (4a vs. 4b) and all predefined covariates were entered simultaneously using a forced-entry approach. The covariates were selected a priori based on prior clinical knowledge and their potential influence on $CD4^{+}$ T-cell recovery, and were retained regardless of their univariable P-values to ensure adequate adjustment for clinically and biologically relevant confounders. This approach was used to avoid excluding important clinical covariates solely on the basis of statistical thresholds. To avoid ambiguity, we have clarified this point in the revised Methods section **(Page 11-12, Lines 444-447)**.

**Comment 16.** Line 393: Statement needs to be supported by a reference. Also, how was the adjustment done?

**Response:** Thank you for your comment. The covariates included in the adjusted models were selected a priori based on prior clinical knowledge and previously reported factors associated with $CD4^{+}$ T-cell recovery in people living with HIV. We have now added the appropriate references to support the selection of these potential confounders **(Page 11-12, Lines 444-450)**.

We have also revised the Methods section to clarify how the adjustment was performed. Specifically, that CRF01_AE cluster 4 subgroups (4a vs. 4b) and all predefined covariates were simultaneously entered into the multivariable regression models using a forced-entry approach. **(Page 11-12, Lines 444-447)**.

**Comment 17.** Line 406: Define and describe details for PSM model

**Response:** Thank you for your comment. We agree that the details of the PSM model were not sufficiently described in the original Methods section. We have now revised this section to provide additional details on the PSM procedure.

Specifically, propensity scores were estimated using a logistic regression model with CRF01_AE cluster 4 subgroups as the dependent variable and baseline $CD4^+$ T-cell count and age at ART initiation as covariates. These two variables were included because they showed substantive baseline imbalance between cluster 4a and cluster 4b before matching ($P < 0.05$ and standardized mean difference [SMD] $>0.1$). One-to-one nearest-neighbor matching without replacement was then performed using the MatchIt package in R. **(Page 12, Lines 457-462)**.

**Comment 18.** Line 411: Using what packages?

**Response:** Thank you for your reminder. We have revised the Methods section to specify the R packages used for the statistical analyses. Propensity score matching (PSM) was performed using the MatchIt package. Survival analyses, including Kaplan–Meier analysis and time-dependent Cox models, were performed using the survival package. Generalized additive mixed models (GAMM) were fitted using the mgcv package. Multivariable logistic regression analyses were performed using the base R stats package. These details have been added to the revised Methods section **(Page 12, Lines 466-469)**.

**Reviewer #3**

**Comment 1**. The absence of baseline HIV RNA levels represents a major limitation that directly affects interpretation of immune recovery. Baseline viral load is one of the strongest predictors of CD4 recovery after ART and may differ substantially between viral lineages. Without adjustment for this parameter, it remains unclear whether the observed differences between cluster 4a and 4b reflect viral pathogenicity or differences in disease stage at ART initiation. Although the authors acknowledge this limitation, the impact is likely underestimated. At present, the central conclusion that cluster 4a is intrinsically more pathogenic remains insufficiently supported.

**Response:** Thank you for this important and insightful comment. We fully agree that baseline HIV RNA level is an important predictor of $CD4^+$ T-cell recovery after ART and that the absence of adjusting baseline viral load data may affect the interpretation of immune reconstitution differences between cluster 4a and cluster 4b.

In our cohort, most participants were enrolled in 2017–2018. At that time, routine baseline HIV RNA testing before ART initiation was not yet universally required in the national treatment workflow. The requirement for baseline viral load testing was formally emphasized in the National Free Antiretroviral Treatment Manual (2023 edition) in China. Therefore, baseline HIV RNA data were missing for a substantial proportion of patients in this study in the national database established under China's National Free Antiretroviral Treatment Program.

To address this limitation as much as possible, we further searched the institution biobank for available baseline plasma samples from the study participants and performed additional HIV RNA testing. Finally, baseline viral load data were obtained for 355 participants. We then conducted additional sensitivity analyses restricted to participants with available baseline HIV RNA data, in which baseline viral load was included as an additional covariate. After further adjustment for baseline viral load, the main findings remained unchanged, suggesting that the association between cluster 4a infection and poorer immune reconstitution was not fully explained by baseline viral load differences. The detailed results of these sensitivity analyses are provided in "Sensitivity analysis" subsection in the Result section **(Page 6, Lines 189-198) (Supplementary Tables S5-S6 and Figure S5)**.

We also agree that baseline disease stage at ART initiation is a critical issue. In addition to viral load, baseline $CD4^+$ T-cell count is a clinically important marker of immune status and cumulative immune damage at ART initiation. In our study, patients infected with cluster 4a had lower baseline $CD4^+$ T-cell counts than those infected with cluster 4b, suggesting potentially more advanced immune impairment at treatment initiation.

Therefore, we used propensity score matching (PSM) to balance baseline CD4$^+$ T-cell count at ART initiation between the two groups. After matching, baseline CD4$^+$ T-cell counts were well balanced between cluster 4a and cluster 4b. Importantly, cluster 4a remained significantly associated with poorer immune reconstitution after PSM, supporting the robustness of our main findings.

Nevertheless, we fully acknowledge that the incomplete availability of baseline HIV RNA data limits our ability to completely exclude residual confounding related to baseline viral load and disease stage. These analyses reduce, but do not completely eliminate, the concern that differences in baseline disease status may have influenced the observed association. We have also expanded the Discussion section to explicitly discuss the absence of complete baseline HIV RNA data and its potential impact on interpretation **(Page 8, Lines 302-306)**.

Furthermore, we have revised the Discussion and Conclusion sections to avoid overinterpreting the findings. In particular, we have softened statements suggesting that cluster 4a is "intrinsically more pathogenic" and now describe our results more cautiously **(Page 7, Lines 261-264; Page 9, Line 329-338)**. as showing that cluster 4a infection was associated with poorer immune reconstitution, which may indicate greater pathogenic potential but requires further validation in studies with complete baseline viral load data.

**Comment 2.** The authors propose that the poorer immune recovery in cluster 4a is "likely driven" by higher CXCR4 tropism. However, CXCR4 tropism was not included in multivariable models, mediation analysis was not performed, direct association between tropism and CD4 recovery was not shown, thus the claim that tropism explains immune recovery differences is currently speculative. To strengthen this central hypothesis, some level of experimental validation is strongly encouraged.

**Response:** Thank you very much for this insightful and rigorous comment. We agree that our previous wording suggesting that poorer immune reconstitution in cluster 4a was "likely driven" by higher CXCR4 tropism was too strong for the current evidence and could imply a causal interpretation that is not fully supported by our observational data. We have therefore carefully revised the manuscript to ensure a more cautious and scientifically appropriate interpretation.

Regarding why CXCR4 tropism was not included in the primary multivariable models, we considered viral tropism to be a potential mediator on the pathway between CRF01_AE cluster 4 subgroups and immune reconstitution, rather than a conventional baseline confounder. Under this assumption, simultaneously adjusting for both cluster 4 subgroups and viral tropism in the primary models would estimate the direct effect of

cluster 4 subgroups independent of viral tropism, rather than the overall association between cluster 4 subgroups and immune reconstitution. Therefore, viral tropism was not included as an adjustment covariate in the primary multivariable models.

Nevertheless, to directly address the reviewer's concern regarding the association between viral tropism and immune reconstitution, we performed additional analyses evaluating the relationship between viral tropism and immune reconstitution. These analyses showed that CXCR4 tropism was associated with poorer immune reconstitution. The detailed results have been added to the Result section **(Page 6, Lines 208-213) (Supplementary Tables S7–S8 and Figure S6)**. These findings support the possibility that CXCR4 tropism may contribute to the poorer immune reconstitution observed in cluster 4a, although they do not establish causality.

We also agree that formal mediation analysis would be the most appropriate statistical approach to evaluate the potential pathway of "cluster 4a → CXCR4 tropism → poorer immune recovery." However, genotypic tropism data were not available for the entire cohort, and the incomplete availability of these data limited our ability to conduct a robust mediation analysis. We have explicitly acknowledged the absence of formal mediation analysis as a limitation in the Discussion section **(Page 9, Lines 321-327)**.

In addition, we fully agree that experimental validation would further strengthen this mechanistic hypothesis. Functional assays, such as pseudovirus-based co-receptor usage assays or experiments assessing the effects of V3 amino acid substitutions on CXCR4/CCR5 interactions, would be valuable for determining whether the sequence differences observed in cluster 4a directly contribute to altered co-receptor usage. However, such experiments are beyond the scope of the current epidemiological cohort study. To provide preliminary structural insight into this potential mechanism, we performed exploratory structural analyses to compare the predicted interactions between cluster-specific V3 structures and the CXCR4/CCR5 co-receptors. Although these computational analyses cannot replace functional validation, they provide preliminary evidence for potential differences in receptor–ligand interactions. We have now clearly stated this limitation and identified functional validation as an important direction for future research **(Page 8-9, Lines 311-321)**.

Accordingly, we have revised the relevant statements throughout the manuscript to avoid overinterpretation. We no longer state that poorer immune reconstitution in cluster 4a is "likely driven by" CXCR4 tropism. Instead, we now describe CXCR4 tropism as a potential explanatory factor and state that the poorer immune reconstitution observed in cluster 4a may be partially associated with its higher prevalence of CXCR4 tropism **(Page 7, Lines 261-264; Page 9, Line 329-338)**. This hypothesis is supported

by epidemiological association and exploratory structural analyses, but requires further confirmation through formal mediation analysis and experimental validation.

**Comment 3.** The manuscript repeatedly states that cluster 4a has "greater pathogenic potential" or is "more virulent." However, the study only demonstrates the differences in immune reconstitution after ART, it does not evaluate disease progression pre-ART/mortality/AIDS events/viral replication capacity. The authors should revise language throughout the manuscript.

**Response:** Thank you very much for pointing out this important issue regarding the interpretation of our findings. We agree that terms such as "greater pathogenic potential," "more virulent," or "pathogenicity" may imply evidence of accelerated pre-ART disease progression, increased viral replication capacity, higher AIDS-event risk, or increased mortality, none of which were directly evaluated in the present study.

We have therefore systematically reviewed the manuscript and revised the language throughout to avoid overinterpretation. Specifically, we removed or replaced overstated terms such as "pathogenic potential," "virulence," and "pathogenicity" when referring to the findings of this cohort. The revised manuscript now describes the results more cautiously and confines the interpretation to the outcomes directly supported by our data, namely that cluster 4a infection was associated with poorer immune reconstitution after ART compared with cluster 4b infection.

The corresponding revisions have been made throughout the manuscript, including the Abstract, Results, Discussion, and Conclusion sections.

Re: Spectrum04222-25R1 (**Co-receptor usage in distinct phylogenetic clusters of HIV-1 CRF01_AE is associated with differential immune reconstitution after antiretroviral therapy**)

Dear Prof. Pingmin Wei:

Your manuscript has been accepted, and I am forwarding it to the ASM production staff for publication. Your paper will first be checked to make sure all elements meet the technical requirements. ASM staff will contact you if anything needs to be revised before copyediting and production can begin. Otherwise, you will be notified when your proofs are ready to be viewed.

**Data Availability:** ASM policy requires that data be available to the public upon online posting of the article, so please verify all links to sequence records, if present, and make sure that each number retrieves the full record of the data. If a new accession number is not linked or a link is broken, provide production staff with the correct URL for the record. If the accession numbers for new data are not publicly accessible before the expected online posting of the article, publication may be delayed; please contact ASM production staff immediately with the expected release date.

**Publication Fees:** For information on publication fees and which article types have charges, please visit ourwebsite. We have partnered with Copyright Clearance Center (CCC) to collect author charges. If fees apply to your paper, you will receive a message from no-reply@copyright.com with further instructions. For questions related to paying charges through RightsLink, please contact CCC at ASM_Support@copyright.com or toll free at +1-877-622-5543. CCC makes every attempt to respond to all emails within 24 hours.

**ASM Membership:** Corresponding authors may join or renew ASM membership to obtain discounts on publication fees. Need to upgrade your membership level? Please contact Customer Service at Service@asmusa.org.

**Embargo Policy:** A press release may be issued as soon as the manuscript is posted on theSpectrum Latest Articles webpage. The corresponding author will receive an email with the subject line "ASM Journals Author Services Notification" when the article is available online.

The ASM Journals program strives for constant improvement in our submission and publication process. Please tell us how we can improve your experience by taking this quick Author Survey.

Thank you for submitting your paper to Spectrum.

Sincerely,
Jaime Soria
Editor
Microbiology Spectrum
