# Supplementary material for: Co-receptor usage in distinct phylogenetic clusters of HIV-1 CRF01_AE is associated with differential immune reconstitution after antiretroviral therapy
Source: Microbiol Spectr. 2026 Jul 16;14(8):e04222-25. doi: 10.1128/spectrum.04222-25 (PMC13436356; doi:10.1128/spectrum.04222-25)
Supplement: Supplementary file 2 [file spectrum.04222-25-s0001.docx]

**Supplementary Materials**

**Co-receptor usage in distinct phylogenetic clusters of HIV-1 CRF01_AE is associated with differential immune reconstitution after antiretroviral therapy**

Yangyang Liu^1+^, Jing Lu^3+^, Tao Qiu^3^, Zhiliang Hu^2^, Wei Li^4^, Xiaoshan Li^5^, Guoping Du^6^, Meng Zhao^1^, Hongfei Ma^1^, Pingmin Wei^1*^, Dandan Yang^3*^, You Ge^2*^

1 Department of Epidemiology and Health Statistics, School of Public Health, Southeast University, Nanjing, Jiangsu, China.

2 Department of Infectious Disease, the Second Hospital of Nanjing, Nanjing University of Chinese Medicine, Nanjing, China.

3 Institute of HIV/AIDS/STI Prevention and Control, Jiangsu Provincial Center for Diseases Control and Prevention, Nanjing, China.

4 Department of Quality Management, Children’s Hospital of Nanjing Medical University, Nanjing, China.

5 Nanjing Medical University Affiliated Wuxi People’s Hospital, Wuxi, China.

6 Southeast University Hospital, Southeast University, Nanjing, Jiangsu, China.

***Corresponding author’s information:**

You Ge: 1 KangFu Road, Nanjing, 211113, China

Email: [geyou_521@126.com](mailto:geyou_521@126.com)

Dandan Yang: 172 Jiangsu Road, Nanjing, 210009, China

Email: [yangdan1997@163.com](mailto:yangdan1997@163.com)

Pingmin Wei: 87 Dingjiaqiao Road, Nanjing, 210009, China

Email: [mpw_1963@126.com](mailto:mpw_1963@126.com)

**^+^These authors have contributed equally to the work.**

**This file includes:**

Supplementary Tables S1 to S8

Supplementary Figure S1 to S6

**Description of supplemental tables and figures**

**Supplementary Table S1:** Comparison of the matching variables between two groups after PSM analysis.

**Supplementary Table S2:** The analysis of annual recovery rate of CD4^+^ T-cell counts after PSM analysis.

**Supplementary Table S3:** Differences in the risk of INR between cluster 4a and cluster 4b patients after PSM analysis.

**Supplementary Table S4:** Pol and env PCR amplification primers.

**Supplementary Table S5:** Differences in CD4^+^ T cell counts recovery between cluster 4a and cluster 4b infection in patients with baseline viral load.

**Supplementary Table S6:** Differences in risk of INR between cluster 4a and cluster 4b infection in patients with baseline viral load.

**Supplementary Table S7:** Association of HIV-1 tropism with CD4^+^ T cell counts recovery.

**Supplementary Table S8:** Association of HIV-1 tropism with risk of INR.

**Supplementary Figure S1:** Distinct CRF01_AE clusters identified by phylogenetic tree based on the method of approximate maximum likelihood.

**Supplementary Figure S2:** The flowchart of sample selection in the analyses of immune reconstitution.

**Supplementary Figure S3:** The smooth curve of CD4⁺ T-cell recovery fitted by GAMM after PSM analysis.

**Supplementary Figure S4:** Comparison of the CD4 cell normalization probability between cluster 4a and cluster 4b patients after PSM analysis.

**Supplementary Figure S5:** Association of CRF01_AE clusters with probabilities of CD4 cell normalization after adjusting for baseline HIV-1 viral load.

**Supplementary Figure S6:** Association of HIV-1 tropism with probabilities of CD4 cell normalization.

**Table S1. Comparison of the matching variables between two groups after PSM analysis**

| Variable | CRF01_AE cluster 4 | | P value | SMD |
| --- | --- | --- | --- | --- |
|  | Cluster 4a  (N=213) | Cluster 4b  (N=213) |  |  |
| Baseline CD4 counts at ART initiation | 308.00  (184.00, 456.00) | 314.00  (172.00, 441.00) | 0.958 | 0.032 |
| Baseline age at ART initiation | 31.00  (26.00, 45.00) | 31.00  (26.00, 44.00) | 0.713 | 0.048 |

**Table S2. The analysis of annual recovery rate of CD4^+^ T cell counts after PSM analysis**

| Variable | All the patients  (N = 426) | | Patients with baseline CD4 cell counts< 500 cells/μL (N = 354) | |
| --- | --- | --- | --- | --- |
|  | Coefficient (95%CI) | ^a^*P* value | Coefficient (95%CI) | ^a^*P* value |
| Cluster 4b | 55.91 (50.05, 61.77) | <0.001 | 56.42 (51.66, 61.18) | <0.001 |
| Cluster 4a | 42.09 (36.25, 47.93) | <0.001 | 45.58 (40.86, 50.30) | <0.001 |
| Reference (Cluster 4b) | —— | —— | —— | —— |
| Year* Cluster 4a (test for interaction) | -13.68 (-21.96, -5.40) | 0.001 | -10.77 (-17.47, -4.08) | 0.002 |

Note: ^a^P value was adjusted by age at ART initiation, baseline CD4^+^ T-cell counts, baseline BMI, baseline hemoglobin, time from HIV diagnosis to ART initiation, gender, transmission route, marital status, education background, and STD history.

Abbreviations: GAMM, generalized additive mixed model.

**Table S3. Differences in the risk of INR between cluster 4a and cluster 4b patients after PSM analysis**

| Variable | Classification | All patients (N = 426) | | | | Patients with baseline CD4 counts < 500 cells/μL (N = 354) | | | |
| --- | --- | --- | --- | --- | --- | --- | --- | --- | --- |
|  |  | INR (%) | *P* value | ^a^OR (95%CI) | ^a^*P* value | INR (%) | *P* value | ^a^OR (95%CI) | ^a^*P* value |
| CRF01_AE cluster 4 | Cluster 4b | 28.64 | 0.024 | —— | —— | 35.06 | 0.034 | —— | —— |
|  | Cluster 4a | 38.97 |  | 1.95 (1.18, 3.23) | 0.009 | 46.11 |  | 1.95 (1.17, 3.23) | 0.010 |
| Baseline age | ≤30 | 21.21 | <0.001 | —— | —— | 27.27 | <0.001 | —— | —— |
|  | 31-50 | 41.71 |  | 1.72 (0.92, 3.23) | 0.091 | 48.34 |  | 1.62 (0.87, 3.05) | 0.132 |
|  | >50 | 54.72 |  | 1.66 (0.65, 4.24) | 0.292 | 59.18 |  | 1.52 (0.59, 3.89) | 0.388 |
| Baseline CD4 counts | ≤350 cells/μL | 54.32 | <0.001 | —— | —— | 54.32 | <0.001 | —— | —— |
|  | >350 cells/μL | 6.56 |  | 0.06 (0.03, 0.12) | <0.001 | 10.82 |  | 0.10 (0.05, 0.21) | <0.001 |
| Baseline BMI | <24 | 33.93 | 0.914 | —— | —— | 40.36 | 0.811 | —— | —— |
|  | ≥24 | 33.33 |  | 1.26 (0.68, 2.33) | 0.468 | 41.89 |  | 1.21 (0.65, 2.25) | 0.547 |
| Baseline hemoglobin | <120 g/L | 46.43 | 0.039 | —— | —— | 52.00 | 0.256 | —— | —— |
|  | 120-160 g/L | 35.29 |  | 0.85 (0.32, 2.72) | 0.744 | 41.16 |  | 0.82 (0.30, 2.22) | 0.698 |
|  | >160 g/L | 22.67 |  | 0.62 (0.19, 1.98) | 0.420 | 32.69 |  | 0.68 (0.21, 2.23) | 0.527 |
| Time from HIV diagnosis  to ART initiation | ≤1 months | 38.34 | 0.071 | —— | —— | 42.53 | 0.486 | —— | —— |
|  | >1months | 30.04 |  | 1.31 (0.78, 2.20) | 0.303 | 38.89 |  | 1.34 (0.80, 2.25) | 0.262 |
| Gender | Male | 33.92 | 0.844 | —— | —— | 40.84 | 0.804 | —— | —— |
|  | Female | 32.00 |  | 0.43 (0.13, 1.43) | 0.168 | 38.10 |  | 0.42 (0.13, 1.42) | 0.163 |
| HIV transmission route | HET | 32.67 | 0.784 | —— | —— | 39.29 | 0.766 | —— | —— |
|  | MSM | 34.15 |  | 1.58 (0.81, 3.06) | 0.180 | 41.11 |  | 1.55 (0.80, 3.02) | 0.197 |
| Marital status | Single | 25.76 | <0.001 | —— | —— | 32.08 | <0.001 | —— | —— |
|  | Married | 46.91 |  | 1.41 (0.77, 2.57) | 0.263 | 53.52 |  | 1.46 (0.80, 2.67) | 0.216 |
| Education background | Junior high school or lower | 49.63 | <0.001 | —— | —— | 58.77 | <0.001 | —— | —— |
|  | High school | 31.40 |  | 0.47 (0.24, 0.92) | 0.028 | 37.25 |  | 0.45 (0.23, 0.88) | 0.020 |
|  | College or higher | 22.94 |  | 0.44 (0.22, 0.89) | 0.023 | 28.26 |  | 0.42 (0.20, 0.85) | 0.016 |
| History of STD | Yes | 28.36 | 0.305 | —— | —— | 33.33 | 0.218 | —— | —— |
|  | No/Unknown | 34.82 |  | 1.71 (0.83, 3.49) | 0.144 | 42.09 |  | 1.71 (0.84, 3.48) | 0.143 |
| INSTI Switcher | No | 33.67 | 0.811 | —— | —— | 40.91 | 0.743 | —— | —— |
|  | Yes | 36.00 |  | 0.97 (0.35, 2.64) | 0.947 | 37.50 |  | 0.95 (0.35, 2.57) | 0.919 |

Abbreviations: INR, immunological non-response, ^a^OR, adjusted odds ratio.

**Table S4. Pol and env PCR amplification primers**

| target gene | primers | primers sequences | HXB2 location |
| --- | --- | --- | --- |
| **RT-PCR** |  |  |  |
| *pol* | MAW26 | TTGGAAATGTGGAAAGGAAGGAC | 2028—2050 |
|  | RT21 | CTGTATTTCTGCTATTAAGTCTTTTGATGGG | 3539—3509 |
| *env* | 44F | ACAGTRCARTGYACACATGG | 6954—6973 |
|  | 35R | AGTGCTTCCTGCTGCTCCCA | 7700—7719 |
| **nPCR** |  |  |  |
| *pol* | PRO-1 | CAGAGCCAACAGCCCCACCA | 2147—2166 |
|  | RT20 | CTGCCAGTTCTAGCTCTGCTTC | 3462—3441 |
| *env* | ENV7 | CTGTTAAATGGCAGTCTAGC | 7002—7021 |
|  | ENV8 | CACTTCTCCAATTGTCCCTCA | 7668—7688 |

**Table S5. Differences in CD4^+^T-cell counts recovery between cluster 4a and cluster 4b infection in patients with baseline viral load**

| Variable | | Coefficient | 95%CI | P value |
| --- | --- | --- | --- | --- |
| **GAMM** | |  |  |  |
|  | Cluster 4a | 56.03 | 49.79-62.28 | <0.001 |
|  | Cluster 4b | 60.40 | 51.27-69.53 | <0.001 |
|  | Reference (Cluster 4a) | —— | —— | —— |
|  | Year* Cluster 4b (test for interaction) | 16.19 | 5.66-26.73 | 0.003**^a^** |

Note: ^a^P value was adjusted by age at ART initiation, baseline CD4^+^ T-cell counts, baseline viral load, baseline BMI, baseline hemoglobin, time from HIV diagnosis to ART initiation, gender, transmission route, marital status, education background, and STD history.

Abbreviations: GAMM, generalized additive mixed model.

**Table S6 Differences in risk of INR between cluster 4a and cluster 4b infection in patients with baseline viral load**

| Variable | | INR (%) | P value | ^a^OR (95%CI) | ^a^P value |
| --- | --- | --- | --- | --- | --- |
| CRF01_AE cluster 4 | |  | 0.001 |  |  |
|  | Cluster 4b | 14.29% (18/126) |  | —— | —— |
|  | Cluster 4a | 29.69% (68/229) |  | 2.06 (1.08-3.96) | 0.030 |
| Baseline age | |  | <0.001 |  | 0.188 |
|  | ≤30 | 13.19% (19/144) |  | —— | —— |
|  | 31-50 | 28.85% (45/156) |  | 1.69 (0.79-3.59) | 0.176 |
|  | >50 | 40.00% (22/55) |  | 2.33 (0.82-6.65) | 0.114 |
| Baseline CD4 counts | |  | <0.001 |  |  |
|  | ≤350 cells/μL | 34.29% (84/245) |  | —— | —— |
|  | >350 cells/μL | 1.82% (2/110) |  | 0.04 (0.01-0.17) | <0.001 |
| Baseline viral load | |  | 0.906 |  |  |
|  | ≤10^5^ copies/mL | 24.40% (61/250) |  | —— | —— |
|  | >10^5^ copies/mL | 23.81% (25/105) |  | 0.63 (0.34-1.20) | 0.159 |
| Baseline BMI | |  | 0.425 |  |  |
|  | Underweight or normal | 25.28% (67/265) |  | —— | —— |
|  | Overweight or obese | 21.11% (19/90) |  | 0.84 (0.42-1.65) | 0.605 |
| Baseline hemoglobin | |  | 0.325 |  |  |
|  | <120 g/L | 26.32% (5/19) |  | —— | —— |
|  | 120-160 g/L | 25.72% (71/276) |  | 1.43 (0.42-4.76) | 0.556 |
|  | >160 g/L | 16.67% (10/60) |  | 1.23 (0.29-5.14) | 0.776 |
| Time from HIV diagnosis  to ART initiation | |  | 0.333 |  |  |
|  | ≤1 months | 26.37% (48/182) |  | —— | —— |
|  | >1months | 21.96% (38/173) |  | 1.55 (0.86-2.78) | 0.146 |
| Gender | |  | 0.827 |  |  |
|  | Male | 24.11% (81/336) |  | —— | —— |
|  | Female | 26.32% (5/19) |  | 0.27 (0.07-0.99) | 0.049 |
| HIV transmission route | |  | 0.108 |  |  |
|  | HET | 31.17% (24/77) |  | 0.65 (0.31-1.37) | 0.257 |
|  | MSM | 22.30% (62/278) |  | —— | —— |
| Marital status | |  | <0.001 |  |  |
|  | Single | 16.67% (36/216) |  | —— | —— |
|  | Married | 35.97% (50/139) |  | 1.47 (0.75-2.89) | 0.263 |
| Education background | |  | 0.003 |  |  |
|  | Junior high school or lower | 33.65% (35/104) |  | —— | —— |
|  | High school | 26.92% (28/104) |  | 0.79 (0.39-1.61) | 0.517 |
|  | College or higher | 15.65% (23/147) |  | 0.54 (0.23-1.26) | 0.154 |
| History of STD | |  | 0.573 |  |  |
|  | Yes | 26.98% (17/63) |  | —— | —— |
|  | No/Unknown | 23.63% (69/292) |  | 0.84 (0.41-1.76) | 0.650 |
| INSTI Switcher | |  | 0.906 |  |  |
|  | No | 23.64% (78/330) |  | —— | —— |
|  | Yes | 32.00% (8/25) |  | 2.17 (0.75-6.27) | 0.152 |

Abbreviations: INR, immunological non-response, ^a^OR, adjusted odds ratio.

**Table S7. Association of HIV-1 tropism with CD4^+^ T cell counts recovery**

| Variable | | Coefficient | 95%CI | P value |
| --- | --- | --- | --- | --- |
| **GAMM** | |  |  |  |
|  | CCR5 tropism | 55.30 | 51.00-59.60 | <0.001 |
|  | CXCR4 tropism | 46.22 | 40.58-51.86 | <0.001 |
|  | Reference (CCR5) | —— | —— | —— |
|  | Year* CXCR4 (test for interaction) | -9.15 | -17.97 to -9.15 | 0.043**^a^** |

Note: ^a^P value was adjusted by age at ART initiation, baseline CD4^+^ T-cell counts, baseline BMI, baseline hemoglobin, time from HIV diagnosis to ART initiation, gender, transmission route, marital status, education background, and STD history.

Abbreviations: GAMM, generalized additive mixed model.

**Table S8 Association of HIV-1 tropism with risk of INR**

| Variable | | INR (%) | P value | ^a^OR (95%CI) | ^a^P value |
| --- | --- | --- | --- | --- | --- |
| HIV-1 tropism | |  | <0.001 |  |  |
|  | CCR5 tropism | 31.66% (101/319) |  | —— | —— |
|  | CXCR4 tropism | 67.07% (55/82) |  | 2.99 (1.61-5.57) | 0.001 |
| Baseline age | |  | <0.001 |  |  |
|  | ≤30 | 25.58% (44/172) |  | —— | —— |
|  | 31-50 | 47.25% (86/182) |  | 1.37 (0.73-2.55) | 0.327 |
|  | >50 | 55.32% (26/47) |  | 1.44 (0.54-3.82) | 0.468 |
| Baseline CD4 counts | |  | <0.001 |  |  |
|  | ≤350 cells/μL | 56.02% (149/266) |  | —— | —— |
|  | >350 cells/μL | 5.19% (7/135) |  | 0.05 (0.02-0.12) | <0.001 |
| Baseline BMI | |  | 0.031 |  |  |
|  | Underweight or normal | 41.49% (134/323) |  | —— | —— |
|  | Overweight or obese | 28.21% (22/78) |  | 0.55 (0.29-1.07) | 0.079 |
| Baseline hemoglobin | |  | 0.128 |  |  |
|  | <120 g/L | 53.13% (17/32) |  | —— | —— |
|  | 120-160 g/L | 38.94% (118/303) |  | 1.04 (0.44-2.48) | 0.930 |
|  | >160 g/L | 31.82% (21/66) |  | 1.36 (0.46-4.00) | 0.572 |
| Time from HIV diagnosis  to ART initiation | |  | 0.215 |  |  |
|  | ≤1 months | 4216% (78/185) |  | —— | —— |
|  | >1months | 36.11% (78/216) |  | 1.19 (0.71-1.98) | 0.513 |
| Gender | |  | 0.713 |  |  |
|  | Male | 38.67% (145/375) |  | —— | —— |
|  | Female | 42.31% (11/26) |  | 0.53 (0.17-1.65) | 0.272 |
| HIV transmission route | |  | 0.216 |  |  |
|  | HET | 44.71 (38/85) |  | —— | —— |
|  | MSM | 37.34% (118/316) |  | 0.80 (0.39-1.65) | 0.542 |
| Marital status | |  | <0.001 |  |  |
|  | Single | 29.92% (76/254) |  | —— | —— |
|  | Married | 54.42% (80/147) |  | 1.91 (1.04-3.51) | 0.037 |
| Education background | |  | 0.012 |  |  |
|  | Junior high school or lower | 47.93% (58/121) |  | —— | —— |
|  | High school | 40.87% (47/115) |  | 0.73 (0.38-1.42) | 0.353 |
|  | College or higher | 30.91% (51/165) |  | 0.79 (0.39-1.60) | 0.512 |
| History of STD | |  | 0.196 |  |  |
|  | Yes | 31.82% (21/66) |  | —— | —— |
|  | No/Unknown | 40.30% (135/335) |  | 1.80 (0.89-3.65) | 0.105 |
| INSTI Switcher | |  | 0.046 |  |  |
|  | No | 37.73% (143/379) |  | —— | —— |
|  | Yes | 59.09% (13/22) |  | 1.50 (0.54-4.21) | 0.439 |

Abbreviations: INR, immunological non-response, ^a^OR, adjusted odds ratio

**Supplementary Figure S1.** Distinct CRF01_AE clusters identified by phylogenetic tree based on the method of approximate maximum likelihood.

**Supplementary Figure S2:** The flowchart of sample selection in the analyses of immune reconstitution.

**Supplementary Figure S3**. The smooth curve of CD4⁺ T-cell recovery fitted by GAMM after PSM analysis, shown for total patients **(A)** and for those with baseline CD4⁺ T-cell counts <500 cells/μL **(B)**.

**Supplementary Figure S4.** Comparison of the CD4 cell normalization probability between cluster 4a and cluster 4b patients after PSM analysis. (A) The cumulative probability of achieving CD4 cell normalization in total patients. (B) The factors associated with CD4 cell normalization identified by multivariate time-dependent Cox regression analysis in total patients. (C) The cumulative probability of achieving CD4 cell normalization in patients with baseline CD4^+^ T-cell counts < 500 cells/μL. (D) The factors associated with CD4 cell normalization identified by multivariate time-dependent Cox regression analysis in patients with baseline CD4^+^ T-cell counts < 500 cells/μL

**Supplementary Figure S5:** Association of Cluster with probabilities of CD4 cell normalization after adjusting for baseline HIV-1 viral load. (**A**)The cumulative probability of achieving CD4 cell normalization. (**B**) The factors associated with CD4 cell normalization identified by multivariate time-dependent Cox regression analysis.

**Supplementary Figure S6:** Association of HIV-1 tropism with CD4 cell normalization. (**A**)The cumulative probability of achieving CD4 cell normalization. (**B**) The factors associated with CD4 cell normalization identified by multivariate time-dependent Cox regression analysis.
